# Effects of exercise interventions on EEG alpha power across different populations: a systematic review and meta-analysis

**DOI:** 10.3389/fpsyg.2026.1752573

**Published:** 2026-08-06

**Authors:** Fujie Liu, Yifei Yang, Yingying Cao, Aiping Chi, Xiaoxiong He

**Affiliations:** 1 School of Physical Education, Shaanxi Normal University, Xi'an, China; 2 School of Physics Education, Xi’an University, Xi'an, China

**Keywords:** brain neural activity, EEG power spectrum, exercise intervention, meta-analysis, systematic review

## Abstract

**Objective:**

The objective of this study was to systematically evaluate the to evaluate the effects of exercise interventions on electroencephalogram (EEG) alpha-band power across different populations.

**Methods:**

PubMed, Embase, Scopus, the Cochrane Library, and Web of Science were searched from inception to June 25, 2026. Randomized controlled trials comparing an exercise intervention with a control condition and reporting EEG alpha-band power were included. Standardized mean differences were calculated as Hedges’ g. Random-effects models using restricted maximum likelihood estimation and Knapp-Hartung adjustment were applied. Heterogeneity, prediction intervals, subgroup effects, intervention-duration meta-regression, influence diagnostics, recording-condition sensitivity analyses, and small-study effects were examined.

**Results:**

Thirteen randomized controlled trials involving 497 participants were included. Exercise interventions were associated with greater EEG alpha-band power than control conditions (Hedges’ g = 1.23, 95% CI [0.44, 2.02], *p* = 0.005), although heterogeneity was considerable (*I^2^* = 91.56%, τ^2^ = 1.49) and the 95% prediction interval included the null value (−1.55 to 4.01). No significant moderation effects were observed for alpha-power metric, population type, or exercise paradigm, and intervention duration was not associated with effect size in chronic-exercise studies (*β* = 0.103, *p* = 0.142). Leave-one-out analyses yielded consistently positive results. The pooled estimate remained positive and statistically significant when the analysis was restricted to studies with clearly defined resting-state conditions (Hedges’ g = 1.21, 95% CI [0.70, 1.72], *p* < 0.001) and reduced heterogeneity (*I^2^* = 69.46%). Egger’s test was not significant (*p* = 0.465), though publication bias could not be excluded. Confidence in the evidence was limited by risk-of-bias concerns, inconsistency, and imprecision.

**Conclusion:**

Exercise interventions were associated with greater EEG alpha-band power on average; however, the considerable heterogeneity and prediction interval crossing the null indicate substantial uncertainty across study settings.

**Systematic review registration:**

https://www.crd.york.ac.uk/PROSPERO/myprospero, CRD420251179061.

## 1 Introduction

Lack of physical exercise is acknowledged globally as a significant modifiable health risk factor closely associated with the onset and progression of various non-communicable diseases. The World Health Organization and related studies have identified insufficient physical activity as a central issue in contemporary public health, with approximately 27.5% of the global population failing to meet the recommended activity levels. In high-income countries, this proportion rises to 36.8% (Chaput et al., 2020; Guthold et al., 2018). The World Health Organization further recommends that adolescents engage in at least 60 min of moderate-to-vigorous physical activity (MVPA) daily to promote holistic development of physical and mental well-being (Hallal et al., 2012). Physical activity levels among university students globally are experiencing a notable decline (Chen et al., 2020). Beyond its established physical health benefits, exercise has been increasingly investigated as a nonpharmacological approach for supporting cognitive and psychological functioning. Previous systematic reviews have reported that exercise may benefit executive function, attention, memory, and other cognitive outcomes across children, adults, and older populations, although the magnitude of these effects varies according to participant characteristics and intervention design (Contreras-Osorio et al., 2021; Northey et al., 2018).

Electroencephalography (EEG) provides a noninvasive method for examining the temporal and oscillatory characteristics of brain activity and is therefore frequently used to investigate the neurophysiological responses associated with exercise. Frequency-domain EEG analysis quantifies oscillatory activity within predefined frequency ranges. Among these oscillations, alpha activity is commonly examined within an approximate frequency range of 8–13 Hz, although the precise boundaries vary across studies and may also be defined according to an individual alpha frequency. Alpha-band activity has been associated with cortical excitability, sensory gating, attentional allocation, and the functional coordination of distributed neural systems (Jensen and Mazaheri, 2010; Klimesch, 2012; Sauseng et al., 2009). However, the functional meaning of increased or decreased alpha-band power is context dependent and may vary according to scalp location, resting or task conditions, whether recordings are obtained with the eyes open or closed, task demands, and the baseline neurophysiological characteristics of participants (Barry et al., 2007; Haegens et al., 2014; Laufs et al., 2003). Accordingly, an increase in alpha-band power should not automatically be interpreted as a general improvement in cognitive performance or brain function.

Exercise-related changes in EEG oscillatory activity have been reported in both acute and chronic intervention studies. Acute aerobic exercise has been associated with changes in postexercise alpha-band activity and electrophysiological indices of attention (Du Rietz et al., 2019; Huang et al., 2018; Moraes et al., 2007), whereas longer-term exercise interventions, including aerobic exercise, Tai Chi, yoga, Pilates, and horseback-riding exercise, have also been examined in healthy and clinical populations (Cho, 2017; Kim et al., 2015; Nagendra et al., 2015; Son and Lim, 2017; Wang et al., 2025; Wang and Lyu, 2024). Nevertheless, findings across individual studies have been inconsistent. This inconsistency may reflect differences in participant age and health status, exercise modality and dose, acute versus chronic intervention designs, the timing of postexercise assessment, and EEG recording and analysis procedures.

Several reviews have previously synthesized evidence concerning exercise and EEG activity. Gramkow reviewed the effects and methodological characteristics of exercise-intervention studies using resting-state EEG and highlighted substantial inconsistency in study quality and EEG analysis procedures (Gramkow et al., 2020). Hosang conducted a broader systematic review of exercise-related changes in EEG-recorded neural oscillations and documented considerable variation in exercise paradigms, recording conditions, and oscillatory outcomes (Hosang et al., 2022). Rodríguez-Serrano reviewed changes in EEG activity and cognition associated with physical activity specifically among older adults (Rodríguez-Serrano et al., 2024), whereas Li et al. (2024) summarized potential oscillatory mechanisms through which physical exercise may influence brain plasticity. Collectively, these reviews demonstrate growing interest in exercise-related modulation of neural oscillations, while also emphasizing the methodological heterogeneity of the available EEG literature.

Despite these contributions, the quantitative evidence concerning the effect of exercise interventions specifically on EEG alpha-band power remains uncertain. Previous reviews were primarily qualitative or narrative, focused on particular age groups or recording conditions, or included a broad range of EEG outcomes and study designs. Consequently, an updated quantitative synthesis restricted to randomized controlled trials is needed to estimate the average effect of exercise interventions on alpha-band power and to determine the extent to which the findings vary across study and EEG methodological characteristics. In particular, differences between absolute and relative alpha power, healthy and clinical populations, acute and chronic exercise paradigms, and resting and task-based recording conditions require careful consideration.

Therefore, the present systematic review and meta-analysis aimed to evaluate the effects of exercise interventions on EEG alpha-band power across different populations. Specifically, this study sought to: (1) estimate the overall effect of exercise interventions on EEG alpha-band power; (2) assess the extent of between-study heterogeneity and the variability of effects across different study contexts; (3) examine potential moderating effects of alpha-power metrics, population characteristics, and exercise paradigms (acute versus chronic); (4) explore the relationship between intervention duration and effect size in chronic-exercise studies; and (5) evaluate the robustness of the findings through influence diagnostics and sensitivity analyses, including those based on EEG recording conditions.

## 2 Methods

This systematic review and meta-analysis strictly follows the Preferred Reporting Items for Systematic Reviews and Meta-Analyses guidelines (Moher et al., 2009). The study is registered in the PROSPERO systematic review database under the registration number CRD420251179061.

### 2.1 Literature search strategy

This paper underwent a systematic review conducted by two researchers (L. F. J. and Y. Y. F.) through comprehensive literature searches across multiple databases including PubMed, Embase, Scopus, the Cochrane Library, and the Web of Science. The search focused on publicly available research and utilized a combination of database keyword searches, free-text searches, and logical operators (‘AND’ and ‘OR’). The search time range spans from the inception of each database to 25 June 2026, with language restricted to English. Key search terms encompassed various aspects of exercise, brain activity measured through EEG, and power spectrum analysis. Furthermore, manual retrieval of reference lists from relevant studies and reviews was performed. A detailed description of the search methodology is available in Appendix.

### 2.2 Inclusion and exclusion criteria for literature

Two reviewers independently imported articles from multiple databases into EndNote 21 software for duplicate title searches, eliminating any redundant literature. They then assessed abstracts and full-text articles to identify relevant studies based on predetermined inclusion and exclusion criteria. The selection criteria were developed following the PICOS framework (Participants, Intervention, Comparator, Outcomes, and Study Design). The inclusion criteria are outlined in Table 1.

**Table 1:** Qualification standards classified by category.

Category	Qualification
Participants	Human participants of any age, including healthy/general and clinical/special populations, who were able to complete the prescribed exercise intervention.
Intervention	A structured acute exercise session or chronic exercise programme (including aerobic exercise, resistance training, mind–body exercises, etc.)
Comparator	No exercise, usual activity, waiting list, maintenance of normal daily routines, or another condition without the target exercise intervention.
Outcomes	Quantitative scalp EEG alpha-band power, including absolute, relative, or transformed alpha-power measures.
Study design	Randomised Controlled Trial (RCT)

Exclusion criteria were as follows: (1) studies in which the intervention did not constitute a structured physical exercise intervention; (2) studies in which participants were unable to complete the prescribed exercise intervention; (3) non-randomized controlled studies; (4) studies for which the full text was unavailable; and (5) studies that did not report extractable EEG -band power data.

### 2.3 Literature data extraction and risk of bias assessment

Two researchers independently screened the titles and abstracts of relevant literature based on predetermined inclusion and exclusion criteria, excluding irrelevant studies. After reviewing the full texts, a final selection was made. Data extraction was then carried out independently using a predefined Excel spreadsheet. Extracted data included: (1) References (first author, publication year); (2) Participant characteristics (initial sample size, mean age/age range); (3) Experimental design grouping; (4) Intervention details (exercise program, cycle, duration, frequency); (5) Outcome measures. Mean and standard deviation of results were also extracted for calculating combined effect size. Any uncertainties were resolved through consultation with a third researcher.

Risk of bias assessment: Two researchers independently applied the Cochrane risk of bias assessment tool to evaluate study quality (Higgins et al., 2011; Sterne et al., 2019). The assessment includes: (1) Bias arising from the randomization process; (2) Bias due to deviations from intended interventions; (3) Bias due to missing outcome data; (4) Bias in measurement of the outcome; (5) Bias in selection of the reported result. Each standard is assessed according to its risk level, categorised as low risk, some concerns, or high risk. Where disagreement arises, two researchers reach consensus through discussion; should the dispute persist, it is resolved by consulting a third researcher.

### 2.4 EEG-specific data extraction and outcome harmonizations

Because EEG acquisition and frequency-domain analysis can substantially affect spectral estimates, EEG-specific methodological information was extracted for each study. Where available, we recorded: (1) EEG system and channels; (2) sampling rate and impedance; (3) reference scheme; (4) filter settings; (5) artifact handling; (6) recording duration and segmentation; (7) recording condition (resting/task; eyes open/closed); (8) timing of EEG acquisition; (9) spectral estimation method and parameters; (10) alpha-frequency boundaries; (11) electrodes or regions of interest; and (12) alpha-power metric (absolute, relative, or transformed). Unreported items were coded as “not reported,” and no parameters were inferred. Basic characteristics of EEG data extraction are detailed in Table 2 and Appendix.

**Table 2:** Extraction of fundamental characteristics from EEG data.

First author/year	Equipment/channel	Sampling rate	Impedance	Power type
Du Rietz et al. (2019)	DC-coupled recording system/ 62-channel	500 Hz	10kΩ	Absolute
Huang et al. (2018)	NeuroScan Quik-Cap/30-channel	500 Hz	5kΩ	Absolute
Kim et al. (2015)	/	/	/	Relative
Nagendra et al. (2015)	CAP100C /20-channel	512 Hz	5kΩ	Absolute
Rathi et al. (2025)	OPEN BCI V4 2.0/16-channel	/	/	Absolute
Cho (2017)	19-channel	256 Hz	/	Relative
Wang and Lyu (2024)	64-channel	1,000 Hz	50kΩ	Absolute
Wang et al. (2025)	64-channel	1,000 Hz	50kΩ	Absolute
Son and Lim (2017)	PolyG-I System/ 8-channel	/	/	Relative
Sun et al. (2026)	TMSi, Netherlands. SAGA 64-channel	1,000 Hz	5kΩ	Absolute
Nomura et al. (2026)	MCScap-E52, MCS Co.52-channel	1,000 Hz	10kΩ	Absolute
Lei et al. (2026)	32-channel	500 Hz	5kΩ	Absolute
Che et al. (2026)	medical engineering GmbH, Austria.32-channel	500 Hz	5kΩ	Relative

This meta-analysis used published summary data only; raw EEG data were not reprocessed. Accordingly, frequency-band definitions and spectral estimation procedures were retained as reported in the original studies. Given differences in units, regions, and calculation methods, standardized mean differences (Hedges’ g) were used. Absolute and relative alpha power were not converted but analyzed together and compared in prespecified subgroup analyses.

### 2.5 Data statistics

Statistical analysis was conducted using R software (version 4.5.2) and the “metafor” package. Given that the studies included had continuous outcomes and the dimensionalities and calculation methods of EEG alpha power were inconsistent, the standardised mean difference Hedges’ g corrected for small sample bias was utilised as the effect size, with a reported 95% confidence interval. A positive effect size indicates the intervention group outperformed the control group. Due to anticipated heterogeneity in population characteristics, exercise interventions, and EEG measurement methods, all analyses were performed using a random-effects model. The restricted maximum likelihood method was employed to estimate between-study variance, and statistical inference was conducted using the Knapp–Hartung method. Heterogeneity was assessed using Cochran’s Q test, *I^2^*, and τ^2^, alongside reporting prediction intervals for the overall effect. Two-tailed *p* < 0.05 was considered statistically significant.

Utilise sensitivity analysis through one-by-one exclusion, assess diagnostic impact, and evaluate robustness of results using Baujat plot. Conduct subgroup analyses based on type of *α* power index, study population, and exercise paradigm (acute versus chronic). Assess inter-subgroup differences through mixed-effects meta-regression and F-test with Knapp-Hartung correction. Perform sensitivity analysis by sequentially restricting studies to those clearly defining resting state or specifying eyes-open/closed conditions. In chronic exercise studies, conduct exploratory univariate meta-regression with intervention duration as a continuous variable, reporting regression coefficients, residual heterogeneity, and R^2^. Publication bias and small sample effects were assessed using a funnel plot and Egger’s regression test. The funnel plot, with Hedges’ g on the x-axis and standard error of the effect size on the *y*-axis, visually examined whether the distribution of study effects symmetrically surrounded the combined effect. Egger’s regression test, using the standard error of the effect size as a predictor variable, quantitatively evaluated funnel plot asymmetry. A regression test with *p* < 0.05 indicated potential funnel plot asymmetry or small sample effects. Given the limited number of studies and high heterogeneity amongst them in this research, caution is advised in interpreting the results of the funnel plot and Egger’s test, without conclusively determining the presence or absence of publication bias.

### 2.6 Assessment of evidence quality

The certainty of evidence for EEG alpha-band power was assessed using the GRADE approach. As all included studies were randomized controlled trials, the evidence initially started at high certainty and was evaluated across five domains: risk of bias, inconsistency, indirectness, imprecision, and publication bias. Risk of bias was based on overall RoB 2 judgments. Inconsistency considered heterogeneity and prediction intervals. Indirectness assessed relevance to the review question. Imprecision was evaluated using confidence intervals and sample size. Publication bias was examined using funnel plots and Egger’s test. Certainty was rated as high, moderate, low, or very low. Two reviewers conducted the assessment independently, with disagreements resolved by discussion. Reasons for downgrading were recorded in the Summary of Findings table.

## 3 Results

### 3.1 Literature search and screening results

**Figure 1:**
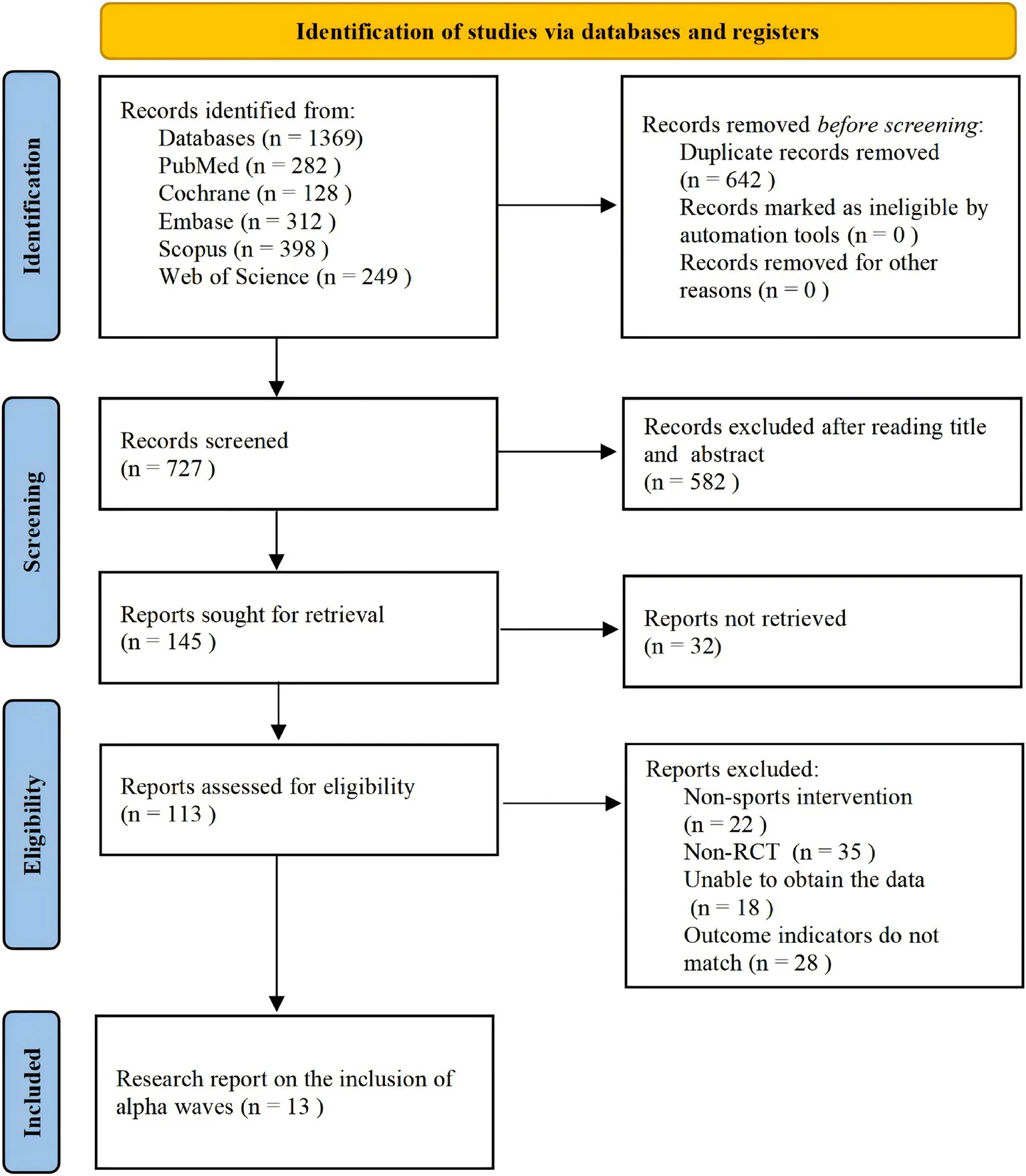
Literature screening flowchart.

A total of 1,369 records were identified through electronic database and manual searches. After 642 duplicate records were removed, the titles and abstracts of the remaining 727 records were independently screened by two reviewers (L. F. J. and Y. Y. F.) against the predefined eligibility criteria. Following title and abstract screening, 582 records were excluded, and 145 full-text reports were assessed for eligibility. Ultimately, 13 studies involving 497 participants were included in the meta-analysis. To assess the reliability of the study-selection process, percent agreement and Cohen’s *κ* were calculated between the two reviewers at both screening stages. Percent agreement was 96.97% during title and abstract screening, with a Cohen’s κ of 0.903, and 95.86% during full-text screening, with a Cohen’s κ of 0.833. According to the criteria proposed by Munoz and Bangdiwala (1997), both κ values indicated almost perfect agreement between the reviewers. Disagreements were resolved through discussion, with a third reviewer (C. Y.) consulted when consensus could not be reached. The study-selection process and screening results are presented in Figure 1.

### 3.2 Basic characteristics included in the study

This meta-analysis included a total of 13 studies (Che et al., 2026; Cho, 2017; Du Rietz et al., 2019; Huang et al., 2018; Kim et al., 2015; Lei et al., 2026; Nagendra et al., 2015; Nomura et al., 2026; Rathi et al., 2025; Son and Lim, 2017; Sun et al., 2026; Wang et al., 2025; Wang and Lyu, 2024). The experimental group consisted of 244 subjects, whilst the control group comprised 253 subjects. The included studies enrolled diverse populations, including healthy participants and individuals with clinical conditions across several age groups, ranging from children and young adults to older adults. The intervention protocols encompassed a wide range of exercise modalities, including aerobic cycling, running, horseback riding, Tai Chi, yoga, Pilates, virtual reality boxing, and high-intensity interval training. The interventions ranged from single acute exercise sessions to programs lasting up to 24 weeks, with individual sessions lasting between 15 and 90 min. All included studies assessed EEG alpha-band activity, with power quantified using absolute or relative measures. EEG recording conditions included resting-state recordings with the eyes open or closed and task-based recordings. The main characteristics of the included studies are summarized in Table 3.

**Table 3:** Basic characteristics of the included literature.

First author/year	Number/age(M ± SD)		Band	Experimental			Control			Intervention programme	Cycle	Time	Frequency/week	Population	Recording_state
	Experimental	Control		N1	Mean change	SD of change	N2	Mean change	SD of change						
Du Rietz et al. (2019)	21.5 ± 2.52	21.5 ± 2.52	α	13	0.223	0.290	13	0.148	0.290	Cycling	acute	30 min	single	Healthy	task
Huang et al. (2018)	9.54 ± 1.59	9.96 ± 1.11	*α*	24	0.100	0.209	28	0.200	0.197	Running	acute	30 min	single	Clinical	resting _open
Kim et al. (2015)	10 69.5 ± 3.2	69.7 ± 3.5	α	10	0.500	0.354	10	−0.500	0.354	Equestrian	8 weeks	15 min	3times	Healthy	resting _close
Nagendra et al. (2015)	22.42 ± 2.30	23.67 ± 2.09	α	15	2.457	0.676	15	−0.052	0.262	Yoga	5 months	90 min	6times	Healthy	resting _close
Rathi et al. (2025)	19.31 ± 1.33	19.8 ± 0.97	α	15	2.875	0.600	19	0.778	0.262	Yoga postures	24 weeks	30 min	2times	Clinical	resting _close
Cho (2017)	67.6 ± 2.32	67.88 ± 2.39	α	15	0.175	0.267	16	−0.171	0.259	Equestrian	12 weeks	25 min	2times	Healthy	resting _close
Wang and Lyu (2024)	20.08 ± 0.28	20.71 ± 0.51	α	35	0.155	0.175	35	−0.681	0.181	Tai Chi	12 weeks	45 min	3times	Healthy	resting _close
Wang et al. (2025)	20.09 ± 0.29	20.70 ± 0.56	α	22	0.155	0.226	23	−0.473	0.218	Tai Chi	12 weeks	45 min	3times	Healthy	resting _close
Son and Lim (2017)	40.81 ± 12.1	36.9 ± 10.71	α	11	−1.615	0.530	10	−0.853	0.358	Pilates	6 weeks	50 min	3times	Clinical	unclear
Sun et al. (2026)	18.00 ± 0.77	18.04 ± 0.84	α	28	0.726	0.207	28	−0.090	0.205	VR boxing	acute	30 min	single	Clinical	resting _unclear
Nomura et al. (2026)	24.2 ± 4.4	25.9 ± 5.5	α	20	0.426	0.281	20	0.048	0.213	Cycling	acute	20 min	single	Healthy	resting _open
Lei et al. (2026)	19.00 ± 1.83	20.19 ± 2.13	α	16	4.000	0.763	16	2.846	0.646	HIIT	8 weeks	30 min	3times	Healthy	resting _close
Che et al. (2026)	21.58 ± 2.21	20.81 ± 2.52	α	20	1.796	0.380	20	1.035	0.287	Cycling	acute	20 min	single	Clinical	resting _unclear

### 3.3 Assessment of bias risk

**Figure 2:**
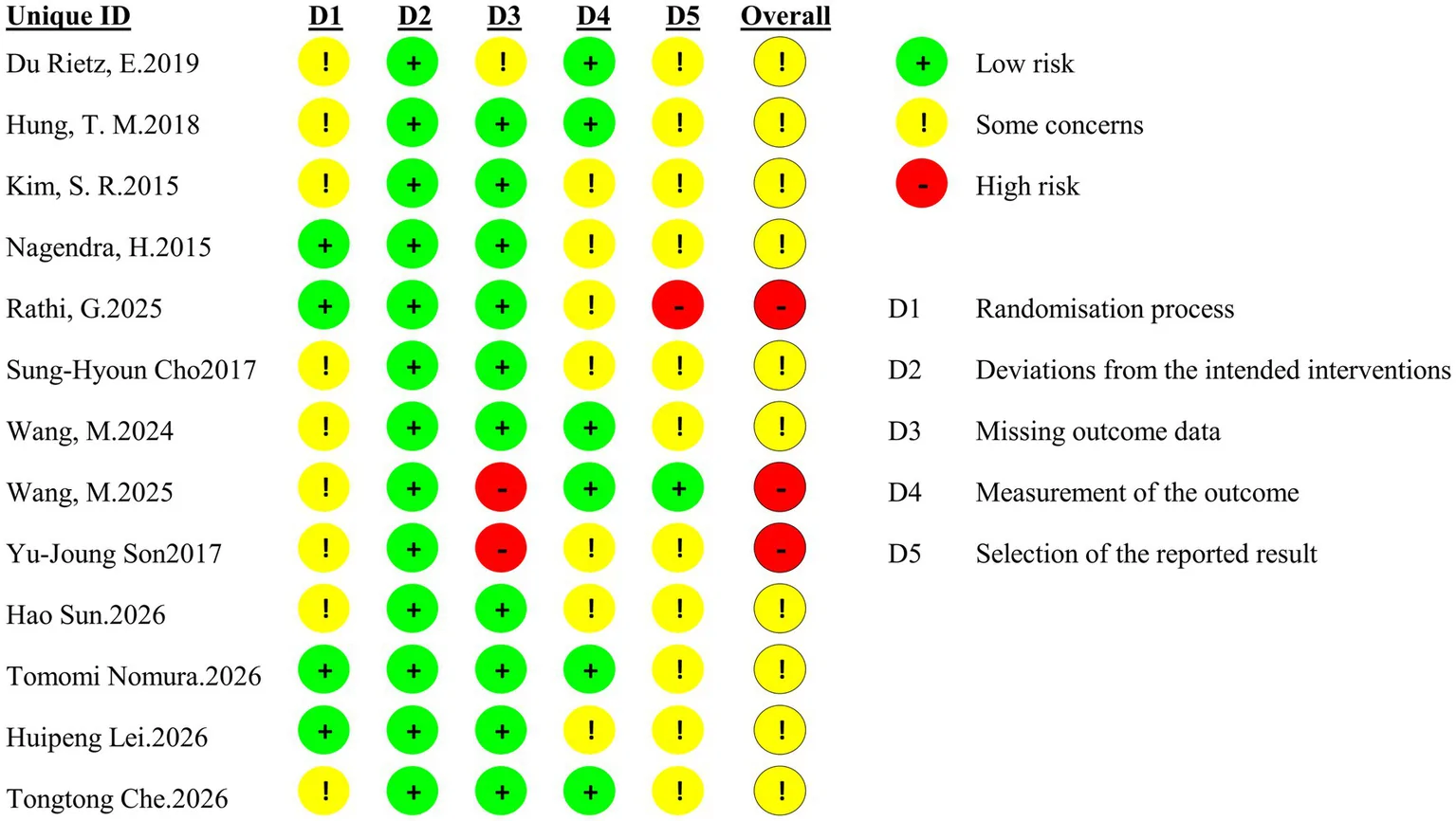
Results of risk of bias for each study.

**Figure 3:**
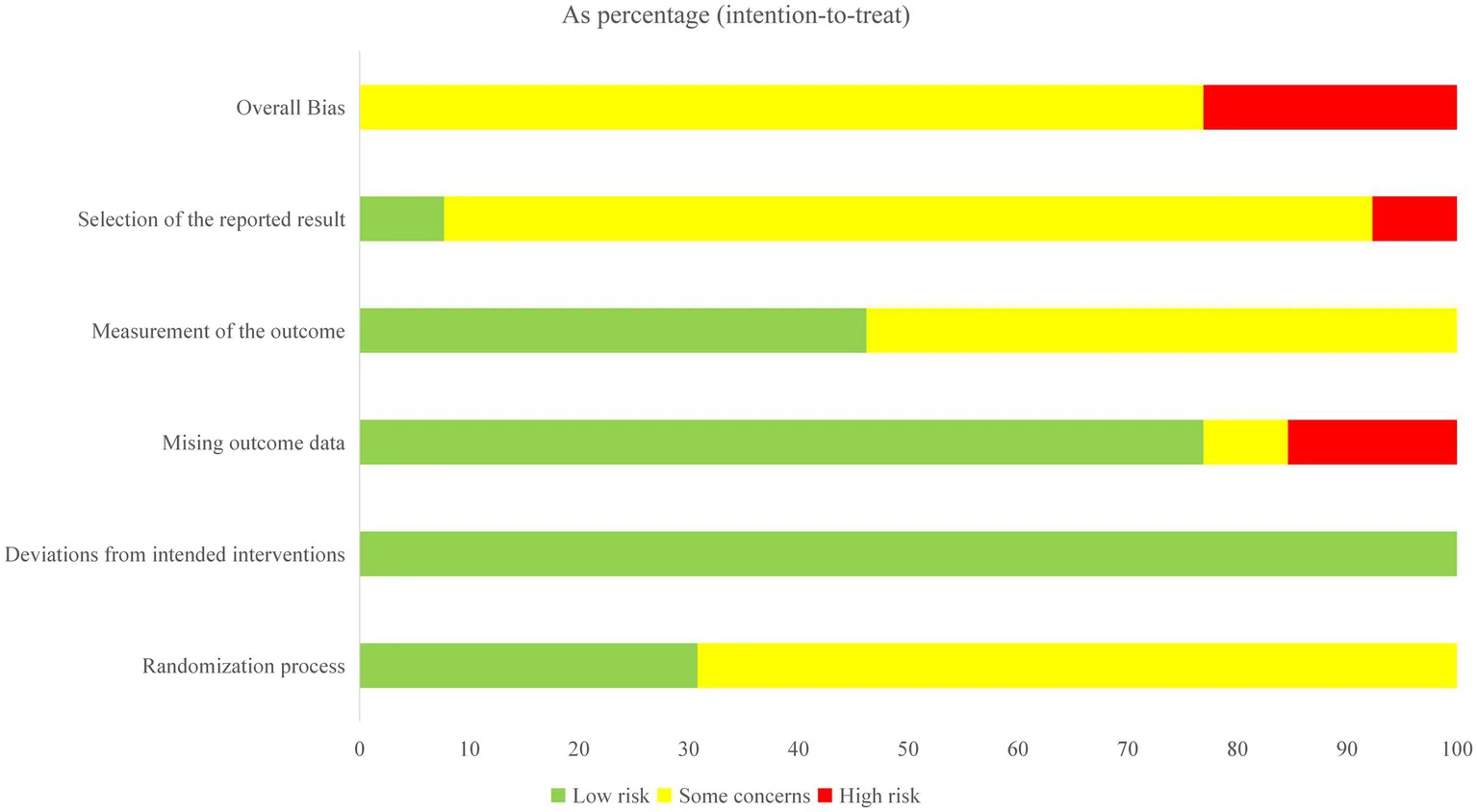
Overall risk of bias results.

Risk of bias was assessed for the EEG alpha-band power result from each of the 13 included randomized trials using the Cochrane Risk of Bias 2 tool. Overall, 10 study results raised some concerns, whereas three were judged to be at high risk of bias. No study result was judged to be at low overall risk of bias. For bias arising from the randomization process, four study results were judged to be at low risk, whereas nine raised some concerns. These concerns primarily reflected insufficient reporting of random sequence generation or allocation concealment; however, none of the study results was judged to be at high risk in this domain. All 13 study results were rated as being at low risk of bias due to deviations from intended interventions. Regarding missing outcome data, 10 study results were judged to be at low risk, one raised some concerns, and two were judged to be at high risk. For bias in measurement of the outcome, six study results were rated as low risk and seven as raising some concerns, with none judged to be at high risk. Bias in selection of the reported result was the most frequent source of concern: one study result was judged to be at low risk, 11 raised some concerns, and one was judged to be at high risk. The three study results judged to be at high overall risk of bias were Rathi et al. (2025), because of a high-risk judgment in the selection-of-the-reported-result domain, and Wang et al. (2025) and Son and Lim (2017), because of high-risk judgments related to missing outcome data. The domain-level and overall risk-of-bias judgments for individual study results are presented in Figure 2, and the distribution of judgments across the five RoB 2 domains is summarized in Figure 3. Detailed signaling-question assessments and supporting justifications are provided in Appendix.

### 3.4 The overall effect of exercise intervention on the alpha power of brain waves

**Figure 4:**
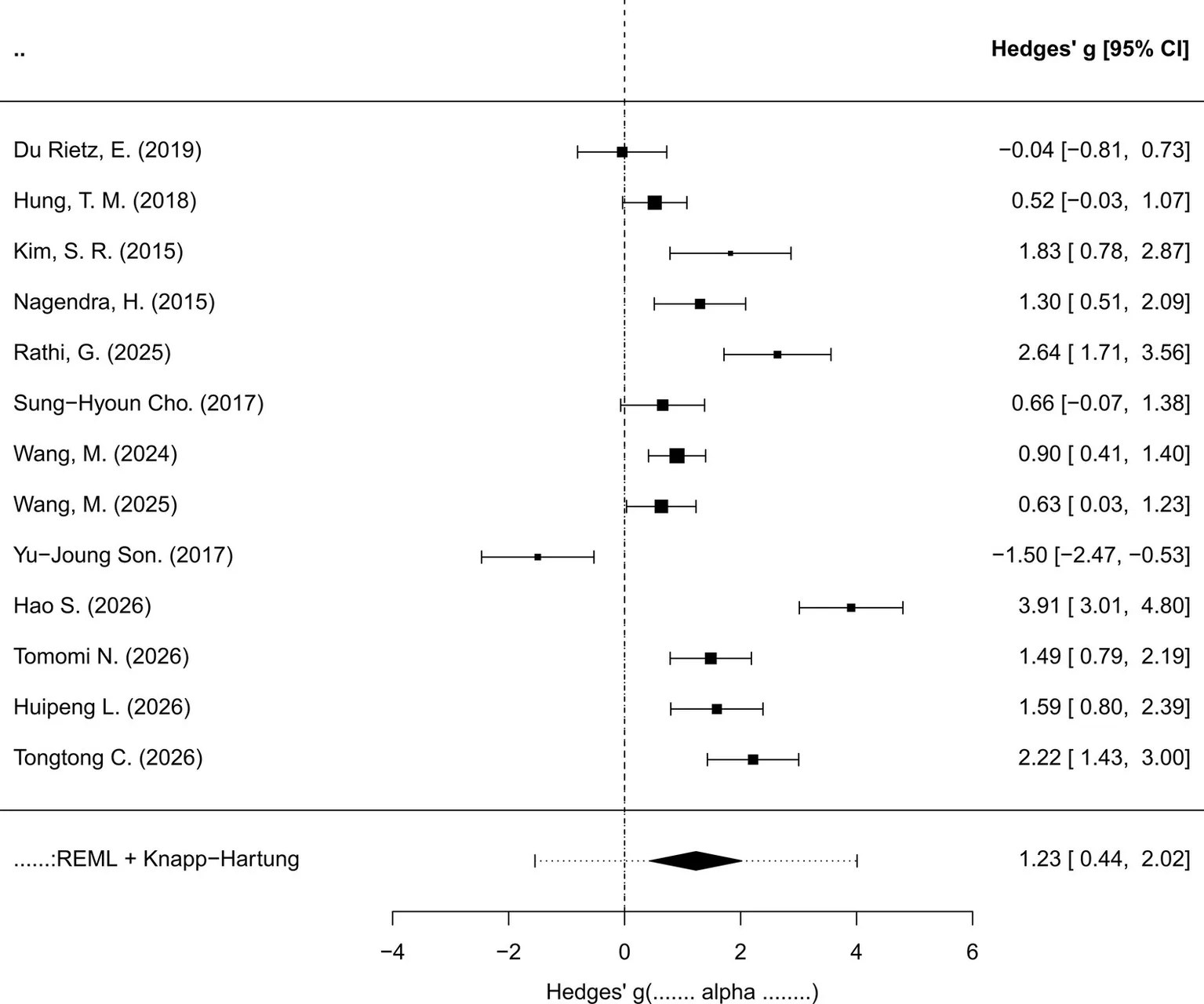
The overall impact of exercise intervention on the alpha power of brain waves.

Thirteen randomized controlled trials involving 497 participants were included, with 244 participants in the experimental groups and 253 in the control groups. Because EEG alpha-band power was reported on different scales across studies, standardized mean differences were calculated as Hedges’ g. Between-study variance was estimated using the restricted maximum likelihood method, and the Knapp-Hartung adjustment was applied to the random-effects model. Considerable between-study heterogeneity was observed (Q = 105.70, df = 12, *p* < 0.001; *I^2^* = 91.56%, τ^2^ = 1.49). The random-effects meta-analysis yielded a statistically significant pooled effect estimate favoring the experimental groups (Hedges’ g = 1.23, 95% CI [0.44, 2.02], t = 3.40, *p* = 0.005). Positive effect sizes were defined as indicating greater increases in EEG alpha-band power in the experimental groups than in the control groups; therefore, the pooled estimate suggests that exercise interventions may increase EEG alpha-band power. However, the 95% prediction interval ranged from −1.55 to 4.01 and included zero, indicating that the true intervention effects may vary considerably across study populations, intervention protocols, and EEG measurement conditions. Accordingly, despite the statistically significant pooled effect estimate, the findings should be interpreted with caution because of the considerable between-study heterogeneity shown in Figure 4.

### 3.5 Exploration of heterogeneity

#### 3.5.1 Subgroup analysis

**Figure 5:**
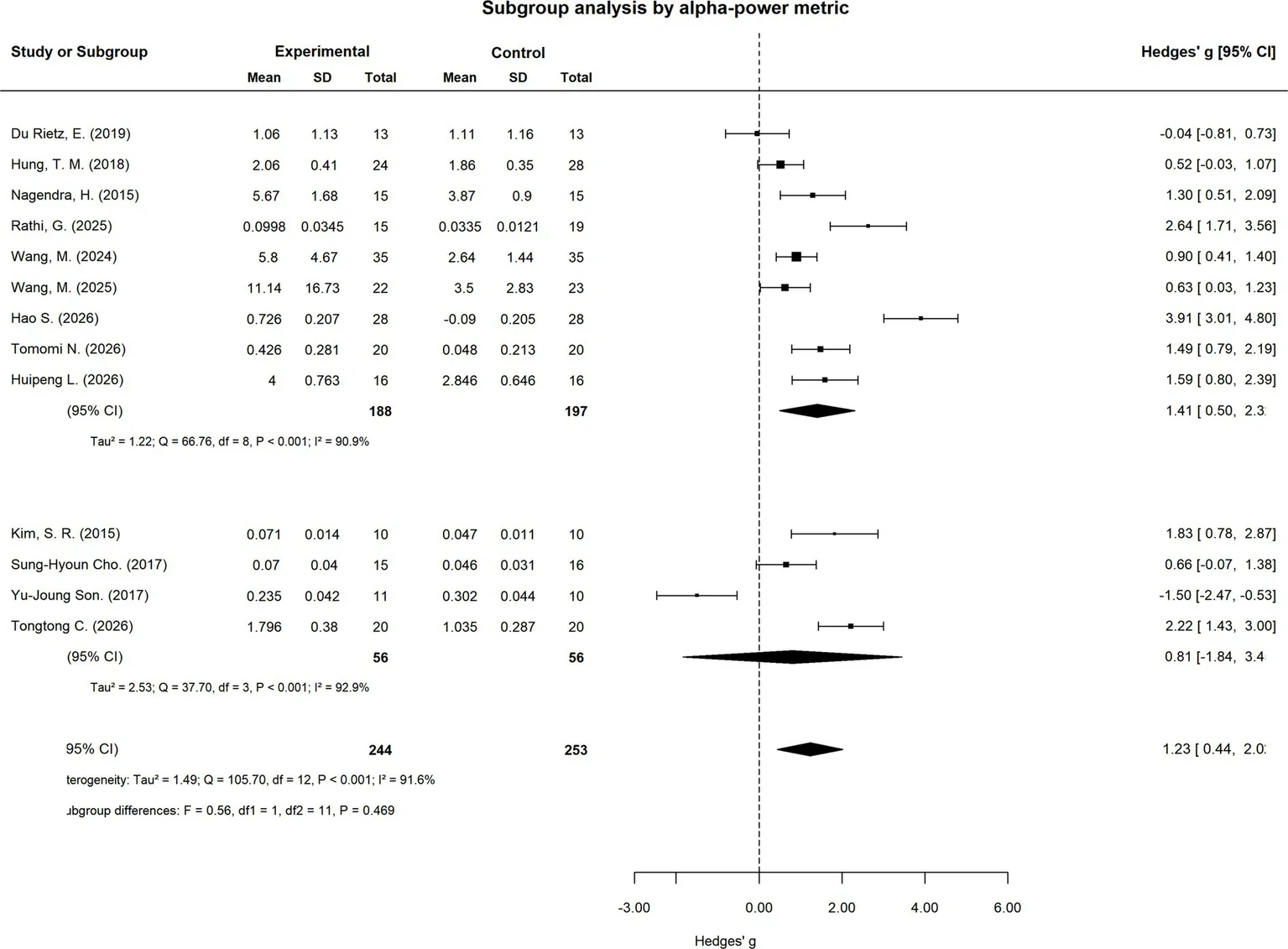
Subgroup analysis of different α power index types.

*α* power indicator type: Among the 13 included studies, nine assessed absolute EEG alpha-band power, comprising 188 participants in the experimental groups and 197 in the control groups, whereas four assessed relative EEG alpha-band power, with 56 participants in each group. In the subgroup of studies assessing absolute EEG alpha-band power, the random-effects analysis yielded a statistically significant pooled effect estimate favoring the experimental groups (Hedges’ g = 1.41, 95% CI [0.50, 2.32], *p* = 0.007). Considerable between-study heterogeneity was nevertheless observed within this subgroup (Q = 66.76, df = 8, *p* < 0.001; *I^2^* = 90.9%, τ^2^ = 1.22). By contrast, the pooled effect estimate for studies assessing relative EEG alpha-band power was not statistically significant (Hedges’ g = 0.81, 95% CI [−1.84, 3.45], *p* = 0.404). Considerable between-study heterogeneity was also observed within the relative-power subgroup (Q = 37.70, df = 3, *p* < 0.001; *I^2^* = 92.9%, τ^2^ = 2.53). Despite the difference in statistical significance between the two subgroup-specific estimates, the mixed-effects analysis showed no statistically significant difference between the absolute- and relative-power subgroups (*p* = 0.469). With absolute EEG alpha-band power as the reference category, the estimated coefficient for relative power was −0.60 (95% CI [−2.37, 1.17]), providing no evidence that the EEG alpha-power metric moderated the effect of exercise interventions on EEG alpha-band power. Consistent with the absence of a significant moderator effect, considerable residual heterogeneity remained after the EEG alpha-power metric was entered into the model (Q_e_ = 104.46, df = 11, *p* < 0.001; residual *I^2^* = 91.98%). The EEG alpha-power metric explained none of the between-study heterogeneity (R^2^ = 0%), indicating that this methodological distinction did not account for the heterogeneity observed in the primary meta-analysis. The results of the subgroup and moderator analyses are presented in Figure 5.

**Figure 6:**
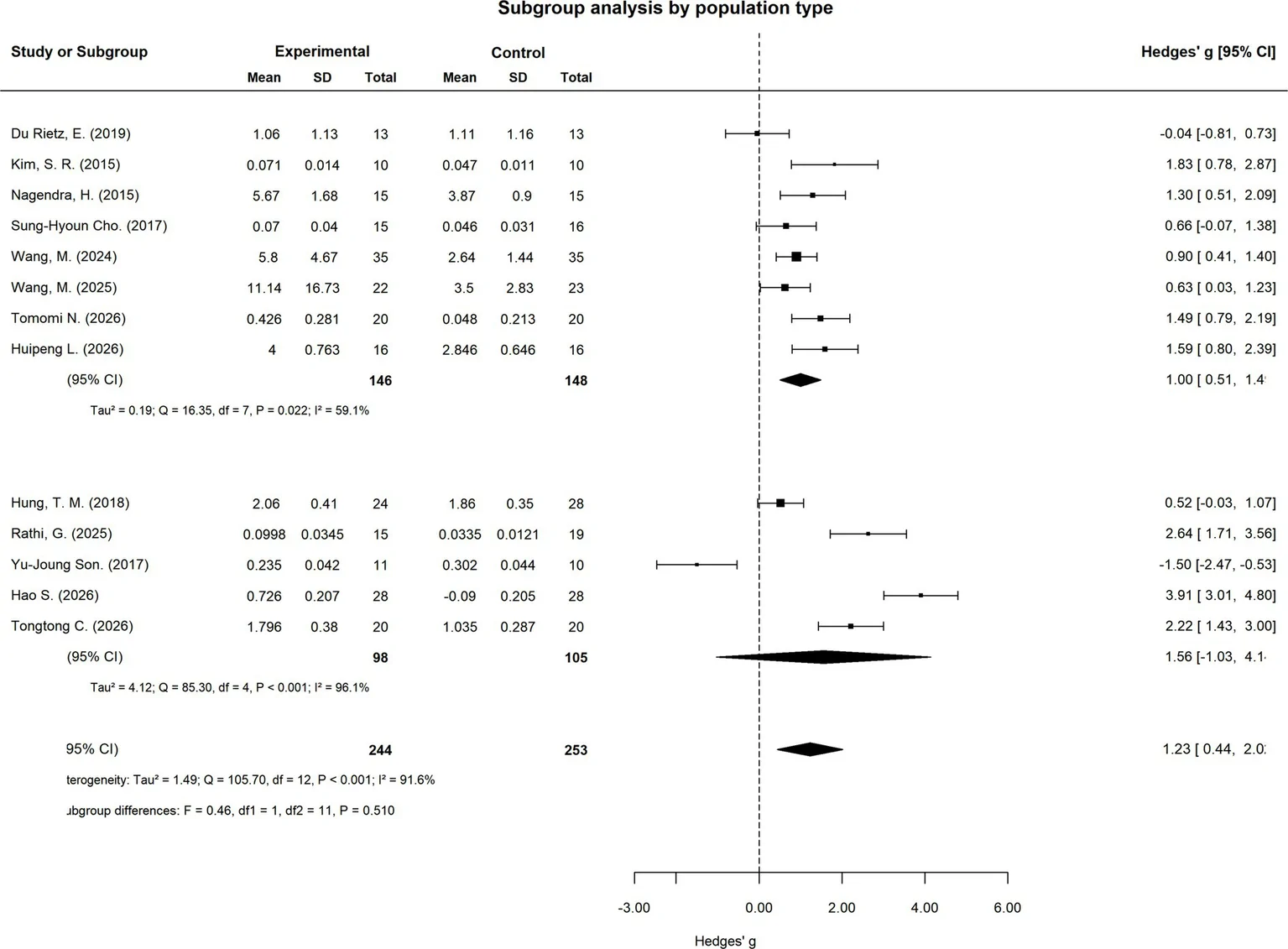
Subgroup analysis based on different types of research subjects.

Type of research subjects: Among the 13 included studies, eight enrolled healthy or general-population samples, comprising 146 participants in the experimental groups and 148 in the control groups, whereas five enrolled clinical or special-population samples, comprising 98 participants in the experimental groups and 105 in the control groups. In the subgroup of studies involving healthy or general-population samples, the random-effects analysis yielded a statistically significant pooled effect estimate favoring the experimental groups (Hedges’ g = 1.00, 95% CI [0.51, 1.49], *p* = 0.002). Moderate between-study heterogeneity was nevertheless observed within this subgroup (Q = 16.35, df = 7, *p* = 0.022; *I^2^* = 59.1%, τ^2^ = 0.19). By contrast, the pooled effect estimate for studies involving clinical or special-population samples was not statistically significant, although it was numerically larger than that observed for healthy or general-population samples (Hedges’ g = 1.56, *p* = 0.170), and its 95% confidence interval included zero. Considerable between-study heterogeneity was observed within the clinical or special-population subgroup (Q = 85.30, df = 4, *p* < 0.001; *I^2^* = 96.1%, τ^2^ = 4.12). Despite the difference in statistical significance between the two subgroup-specific estimates, the mixed-effects analysis showed no statistically significant difference between the healthy or general-population and clinical or special-population subgroups (*p* = 0.510). With the healthy or general-population subgroup as the reference category, the estimated coefficient for the clinical or special-population subgroup was 0.52 (95% CI [−1.16, 2.20]), providing no evidence that population type moderated the effect of exercise interventions on EEG alpha-band power. The results of the subgroup and moderator analyses are presented in Figure 6.

**Figure 7:**
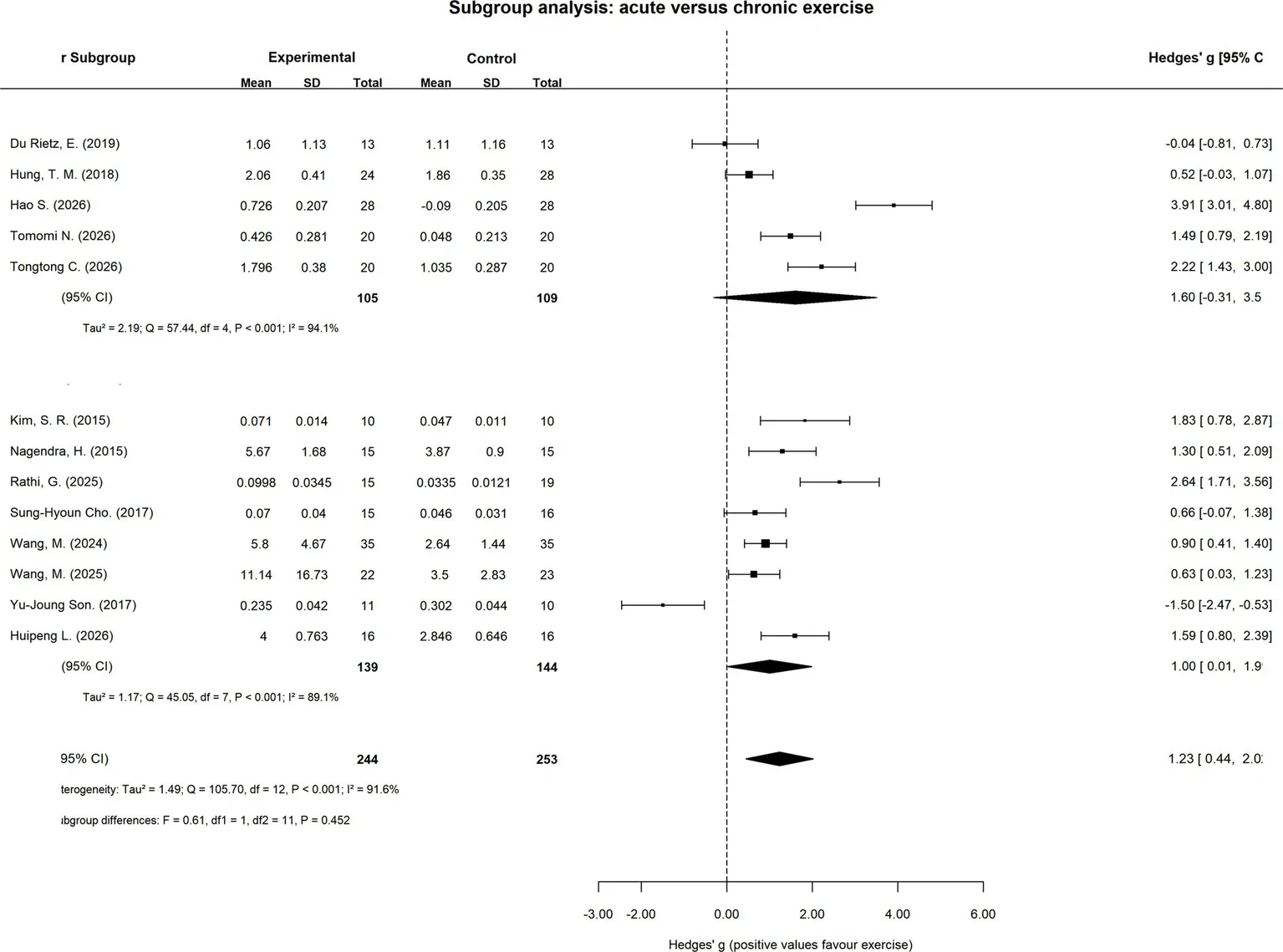
Subgroup analysis of acute exercise and chronic exercise.

Exercise intervention period: The included studies were classified into acute- and chronic-exercise subgroups according to the exercise intervention paradigm. The pooled effect estimate for acute exercise was not statistically significant (five studies; Hedges’ g = 1.60, 95% CI [−0.31, 3.51], *p* = 0.081), whereas the chronic-exercise subgroup yielded a statistically significant pooled effect estimate favoring the experimental groups (eight studies; Hedges’ g = 1.00, 95% CI [0.01, 1.99], *p* = 0.048). Considerable between-study heterogeneity was observed within both subgroups (acute exercise: Q = 57.44, *p* < 0.001, *I^2^* = 94.1%, τ^2^ = 2.19; chronic exercise: Q = 45.05, *p* < 0.001, *I^2^* = 89.1%, τ^2^ = 1.17). Although the pooled effect estimate for acute exercise was numerically larger than that for chronic exercise, the mixed-effects analysis showed no statistically significant difference between the two subgroups (*F* (1, 11) = 0.61, *p* = 0.452). With the acute-exercise subgroup as the reference category, the estimated coefficient for the chronic-exercise subgroup was −0.59 (95% CI [−2.25, 1.07]), providing no evidence that the exercise intervention paradigm moderated the effect of exercise on EEG alpha-band power. Considerable residual heterogeneity remained after the exercise intervention paradigm was entered into the model as a moderator (Q_e_(11) = 102.49, *p* < 0.001; residual *I^2^* = 91.73%), and the moderator explained none of the between-study heterogeneity (R^2^ = 0%). Taken together, these findings provide no evidence that the effects of acute and chronic exercise on EEG alpha-band power differ significantly or that the exercise intervention paradigm moderates the pooled effect estimate. The results of the subgroup and moderator analyses are presented in Figure 7.

#### 3.5.2 Exploratory meta-regression on the duration of chronic exercise interventions

**Figure 8:**
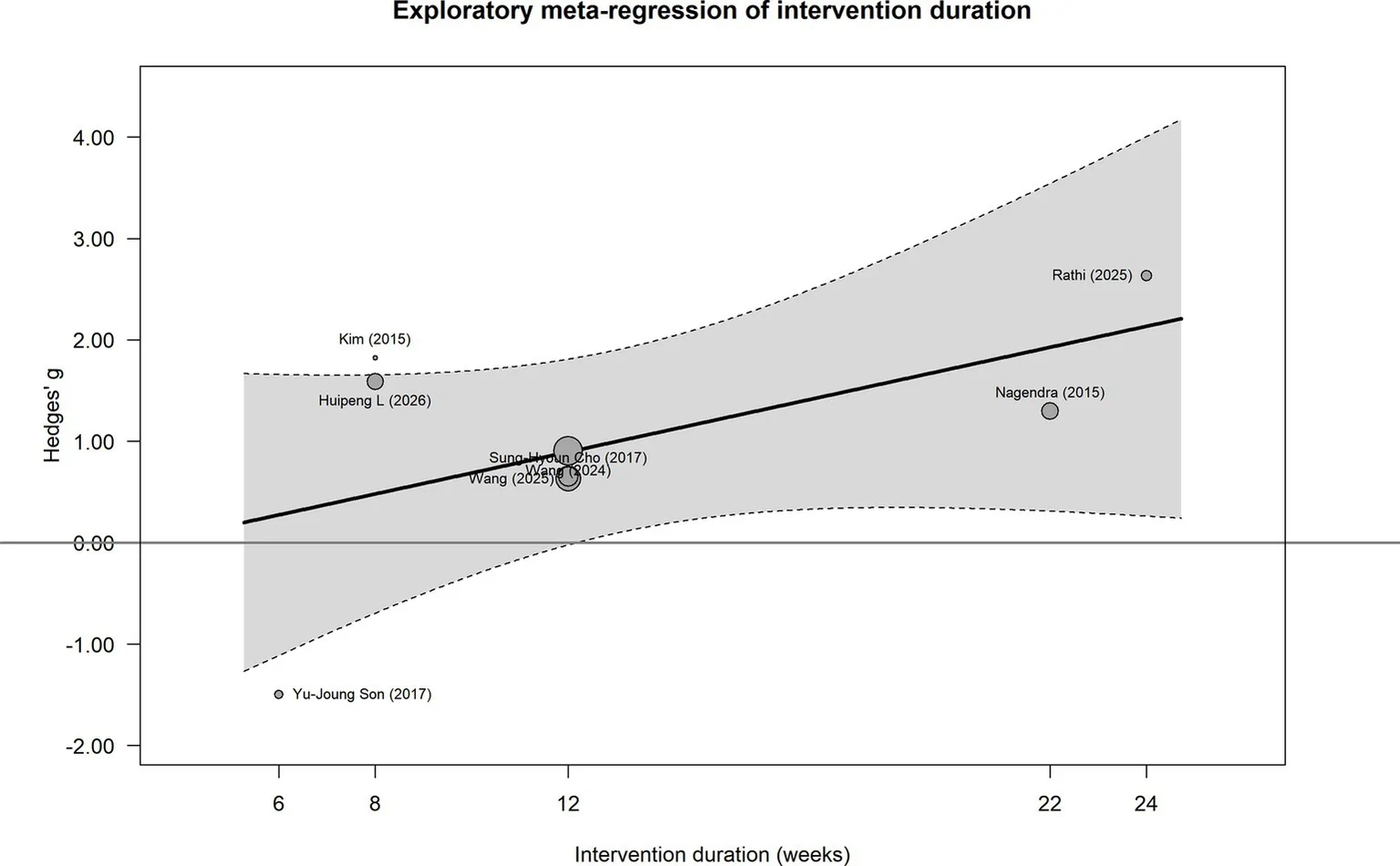
Meta-regression analysis of the duration of chronic exercise intervention.

Because intervention duration in weeks was applicable only to studies of chronic exercise, an exploratory univariable meta-regression was conducted using the eight studies in this subgroup. Intervention duration ranged from 6 to 24 weeks, with a mean of 13 weeks. Although the estimated effect size increased by 0.103 units for each additional week of intervention, there was no statistically significant linear association between intervention duration and effect size (*β* = 0.103, 95% CI [−0.046, 0.253]; *F* (1, 6) = 2.86, *p* = 0.142). Intervention duration accounted for approximately 26.81% of the between-study heterogeneity (R^2^ = 26.81%). Nevertheless, considerable and statistically significant residual heterogeneity remained (residual *I^2^* = 86.18%; Q_e_(6) = 31.29, *p* < 0.001). Given that only eight studies were included in the meta-regression, statistical power was limited, and the estimated association may have been sensitive to the influence of individual studies (Figure 8). Accordingly, these findings should be regarded as exploratory and should not be interpreted as evidence that longer exercise interventions cause greater increases in EEG alpha-band power.

### 3.6 Sensitivity analysis and impact diagnosis

#### 3.6.1 Eliminate one by one the analysis and the impact assessment

Given the considerable heterogeneity observed in the primary meta-analysis, a leave-one-out sensitivity analysis was conducted by excluding each study in turn. The pooled effect estimates remained positive and statistically significant across all iterations and ranged from 1.01 to 1.44, supporting the stability of the direction and statistical significance of the pooled effect estimate. However, the *I^2^* values remained considerable across the leave-one-out analyses, ranging from 87.51 to 92.43%, suggesting that the between-study heterogeneity was not attributable to any single study. Influence diagnostics revealed that Sun et al. (2026) and Son and Lim (2017) had relatively large studentized residuals and Cook’s distances, indicating that these studies had a greater influence on the pooled effect estimate and the estimated between-study variance than did the remaining studies. Nevertheless, neither study was considered sufficiently influential to warrant exclusion. Accordingly, because no data extraction errors or methodological inconsistencies were identified, neither study was excluded solely for the purpose of reducing between-study heterogeneity.

#### 3.6.2 Analysis of EEG recording state sensitivity

**Figure 9:**
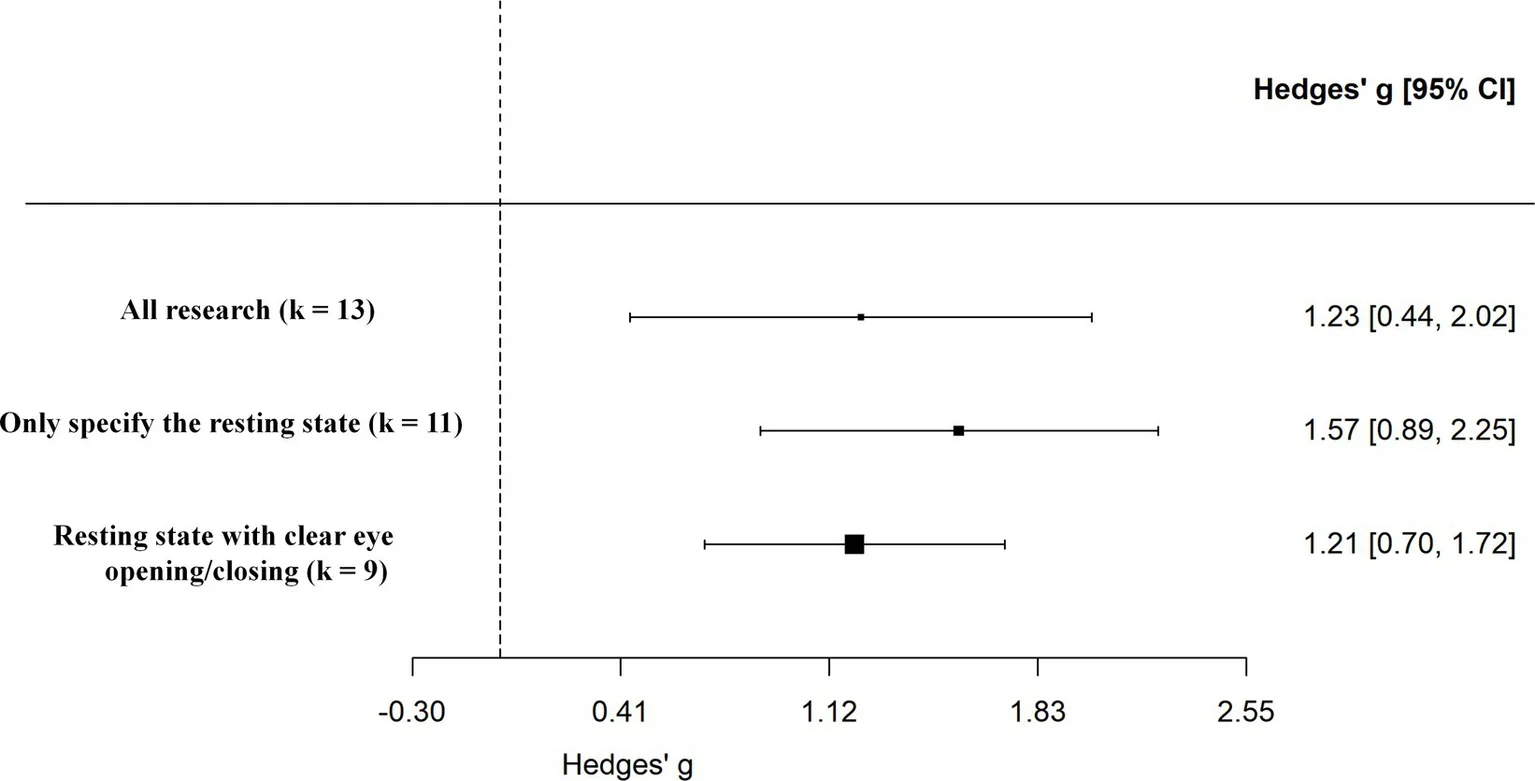
Analysis of EEG recording state sensitivity.

To examine the influence of EEG recording states on combined outcomes, a sensitivity analysis was conducted. When all 13 studies were included (Hedges’ g = 1.23, 95% CI [0.44, 2.02], *p* = 0.005), there was high heterogeneity amongst studies (*I^2^* = 91.56%, τ^2^ = 1.49). After excluding studies with unclear task or recording states, the combined effect size based on 11 studies during rest was 1.57 (95% CI [0.89, 2.25], *p* < 0.001), with heterogeneity reduced to *I^2^* = 86.71% (τ^2^ = 0.86). Further analysis including only 9 studies that explicitly reported eyes-open or eyes-closed rest states showed a statistically significant combined effect size (Hedges’ g = 1.21, 95% CI [0.70, 1.72], *p* < 0.001), with heterogeneity further reduced to *I^2^* = 69.46% (τ^2^ = 0.28) (Figure 9).

These results indicate that under different inclusion criteria, the direction and statistical significance of the combined effect did not substantially change, suggesting robustness in the overall findings of physical exercise improving brain alpha wave power. Moreover, as EEG recording conditions became more consistent, inter-study heterogeneity notably decreased, indicating that recording states may be one of the potential sources of overall heterogeneity.

### 3.7 Report bias and assessment of small sample effect

**Figure 10:**
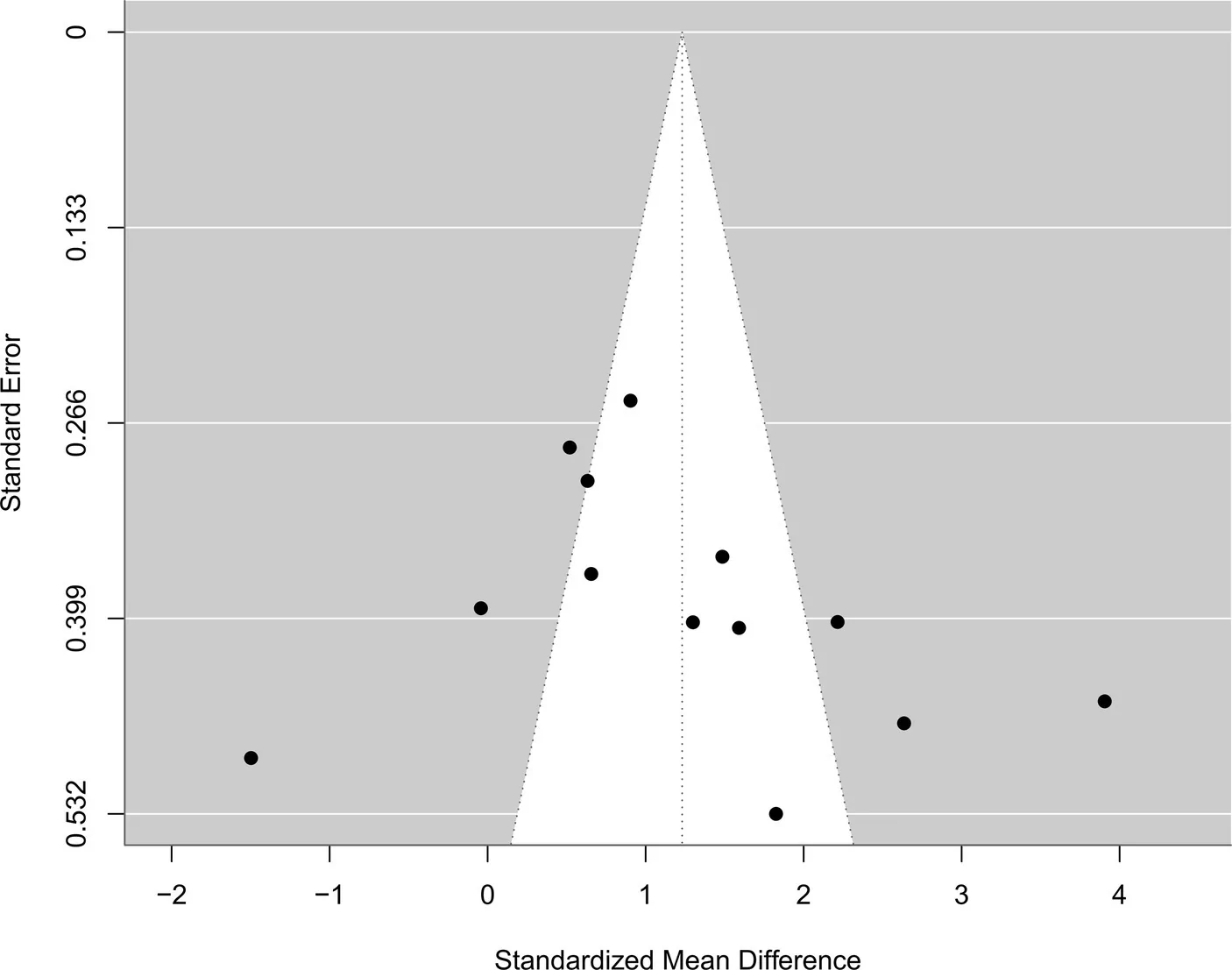
Publication bias funnel plot.

Because 13 studies were included in the meta-analysis, potential publication bias was assessed by visual inspection of the funnel plot and Egger’s regression test. Although visual inspection suggested some funnel plot asymmetry (Figure 10), Egger’s regression test provided no statistically significant evidence of small-study effects (*p* = 0.465). Nevertheless, given the relatively small number of included studies and the considerable between-study heterogeneity, the power of Egger’s regression test was likely limited, and publication bias could not be ruled out. These findings should therefore be interpreted cautiously.

### 3.8 GRADE evidence certainty assessment

The certainty of evidence for the effect of exercise interventions on EEG alpha-band power was rated as very low. Although derived from randomized controlled trials, the evidence was downgraded due to several limitations. Risk of bias was considered serious, as none of the 13 included studies was judged to be at low overall risk; most raised some concerns or were at high risk. Inconsistency was also serious, with substantial heterogeneity observed (I^2^ = 91.56%) and a wide prediction interval crossing the null, indicating variability in effect size across studies. No downgrade was applied for indirectness, as all studies directly addressed the research question. Imprecision was judged to be serious due to the limited number of studies (n = 13), relatively small sample size (n = 497), and a wide confidence interval (Hedges’ g = 1.23, 95% CI [0.44, 2.02]). Publication bias was not formally detected (Egger’s test p = 0.465), although it could not be ruled out. Overall, confidence in the pooled effect estimate was limited. The GRADE assessment is presented in Table 4.

**Table 4:** GRADE summary of findings.

Outcome	No. of studies	No. of participants	Study design	Pooled effect	Risk of bias	Inconsistency
EEG alpha-band power	13	497	Randomized controlled trials	Hedges’ g = 1.23, 95% CI [0.44, 2.02]; 95% prediction interval [−1.55, 4.01]	Serious	Serious

## 4 Discussion

### 4.1 Main findings and the effects of exercise intervention on brainwave activity

The main finding of this study is that exercise interventions may modulate brain electrophysiological activity, as reflected by increases in EEG alpha-band power. This interpretation was further supported by sensitivity analyses, in which the pooled effect estimate remained positive after each study was excluded in turn and after the analysis was restricted to studies with comparable EEG recording conditions. Nevertheless, considerable between-study heterogeneity persisted, and subgroup analyses did not detect statistically significant differences across subgroups defined by the EEG alpha-power metric used, the study population, or whether the intervention involved acute or chronic exercise. Consistent with these subgroup analyses, an exploratory meta-regression restricted to studies of chronic exercise found no evidence of a linear association between intervention duration and effect magnitude. Overall, these findings are consistent with previous evidence that exercise may influence EEG alpha-band activity (Hosang et al., 2022; Shi et al., 2025), although it remains unclear whether the magnitude of this effect varies systematically with the EEG alpha-power metric used, the population studied, or the characteristics of the exercise intervention.

The increase in EEG alpha-band power indicated by the combined analysis may be interpreted in light of the functional roles commonly attributed to alpha activity. Specifically, EEG alpha activity has been implicated in the regulation of cortical excitability (Sauseng et al., 2009), sensory gating, attentional resource allocation, and large-scale neural synchronization (Dumas et al., 2010). Accordingly, exercise-related increases in EEG alpha-band power may reflect a shift toward lower cortical excitability and more efficient neural information processing. This interpretation is broadly consistent with commonly reported psychological responses to exercise, including relaxation, reduced stress, and improved mood (Salmon, 2001; Scully et al., 1998). Although the present meta-analysis focused specifically on alpha-band power, the functional interpretation of EEG activity should be considered within the broader context of neural oscillations across multiple frequency bands. Beta activity has commonly been associated with alertness, sustained attention, and active cognitive processing, whereas theta activity has been linked to memory encoding and emotional processing (Koketsu et al., 2024). Delta activity is most prominent during deep sleep and has been implicated in physiological recovery, whereas gamma activity has been associated with neural information integration and higher-order cognitive processing (Tasali et al., 2008). Against this broader neurophysiological background, the increase in EEG alpha-band power identified in the present meta-analysis may reflect exercise-related changes in functional brain states and neural oscillatory dynamics. This interpretation is also compatible with evidence from previous systematic reviews and meta-analyses showing that regular exercise is associated with improvements in executive function, memory, and attention (Young et al., 2015). For example, an observational study of habitual physical activity found that older adults with higher activity levels showed better cognitive performance and a shift toward faster resting-state EEG rhythms (Sanchez-Lopez et al., 2018). Complementing this observational evidence, experimental findings have also indicated that exercise may modulate the P300 component and theta-band oscillations during inhibitory-control processing (Liu et al., 2025). Taken together, the behavioral and electrophysiological evidence suggests that increased EEG alpha-band power may represent a potential neurophysiological correlate of exercise-related improvements in cognitive function, although its mechanistic role remains to be established.

The potential functional interpretations outlined above should nevertheless be treated with caution. Increased EEG alpha-band power should not be regarded as a general marker of improved cognitive performance or global brain function (Laufs et al., 2003). This is because the functional significance of alpha activity is highly context dependent (Akas et al., 2026). Its functional significance may vary with the recording site (Jensen and Mazaheri, 2010; Klimesch, 2012), whether the eyes are open or closed (Barry et al., 2007), whether participants are at rest or engaged in a task, the specific cognitive demands of that task (Haegens et al., 2014), and participants’ baseline neurophysiological state. Accordingly, the positive pooled estimate should be interpreted as evidence of an exercise-related increase in EEG alpha-band power rather than as evidence of generalized improvement across cognitive domains (Northey et al., 2018). The need for caution is further reinforced by the considerable between-study heterogeneity and the wide prediction interval, which included zero, suggesting that the magnitude—and potentially the direction—of the true effects may vary across study settings. The magnitude of the pooled estimate may also have been influenced by sampling variability in small studies, heterogeneity in EEG outcome definitions and analytic procedures, and a small number of relatively influential studies. The pooled findings should therefore be interpreted in relation to the direction of the effect, the confidence interval around the pooled estimate, the width and position of the prediction interval, and methodological heterogeneity across studies, rather than on the basis of the pooled point estimate alone (Nakagawa and Cuthill, 2007).

### 4.2 Potential moderating factors and heterogeneity in EEG methodology

To explore potential sources of the considerable between-study heterogeneity, three subgroup analyses were prespecified according to the EEG alpha-power metric, population type, and exercise intervention paradigm (acute vs. chronic). EEG acquisition and processing characteristics were also considered qualitatively in an exploratory analysis. None of the corresponding tests for subgroup differences was statistically significant, providing no evidence that the EEG alpha-power metric, population type, or exercise intervention paradigm moderated the effect of exercise interventions on EEG alpha-band power.

#### 4.2.1 Analysis of alpha-power metric types

The pattern of findings appeared to differ according to the metric used to quantify EEG alpha-band power. Studies measuring absolute EEG alpha-band power yielded a statistically significant pooled effect estimate, whereas the pooled estimate for studies measuring relative power was not statistically significant. However, the formal test for subgroup differences was not statistically significant, indicating that this apparent difference should not be interpreted as evidence of a true moderating effect. Absolute power quantifies the magnitude of EEG activity within a specified frequency band and is therefore sensitive to changes in the amplitude of alpha-band oscillations (Cochin et al., 1999; Kim et al., 2017). By contrast, relative power expresses alpha-band power as a proportion of total spectral power and may therefore change as a result of variation in either alpha-band power itself or power in other frequency bands (Clarke et al., 2001; Ergenoglu et al., 2004). For example, if exercise increases both alpha- and beta-band power, absolute alpha power would increase, whereas relative alpha power could remain unchanged or even decrease because total spectral power also increases (Gasser et al., 1988). This measurement dependence suggests that absolute and relative power may differ in their sensitivity to exercise-related changes in alpha-band activity. These considerations have implications for the selection and reporting of EEG alpha-power metrics in future studies. However, because relatively few studies reported relative power, the subgroup comparison had limited statistical power, and the available evidence is insufficient to determine whether the EEG alpha-power metric is a true moderator of the exercise-related effect.

#### 4.2.2 Analysis of the types of research subjects

With respect to population type, studies involving healthy or general-population samples yielded a statistically significant positive pooled effect estimate, with moderate within-subgroup heterogeneity. By comparison, heterogeneity was descriptively lower in this subgroup than in the clinical or special-population subgroup (Dale et al., 2025). The clinical or special-population subgroup yielded a numerically larger pooled point estimate; however, its confidence interval was substantially wider, and considerable within-subgroup heterogeneity was observed (Lai et al., 2019). Importantly, the formal test for subgroup differences was not statistically significant, providing no evidence that population type moderated the effect of exercise interventions on EEG alpha-band power. The clinical or special-population subgroup was also clinically heterogeneous, encompassing participants with attention-deficit/hyperactivity disorder, psychiatric conditions, depressive symptoms, primary dysmenorrhea, and problematic smartphone use. Clinical populations may differ substantially in baseline neurophysiological activity, symptom severity, medication use, cognitive functioning, and exercise tolerance. Although combining these heterogeneous populations into a single subgroup increased the number of studies available for analysis, it may also have obscured variation in exercise-related responses associated with specific clinical characteristics. Accordingly, the considerable heterogeneity observed within the clinical or special-population subgroup may partly reflect differences in underlying conditions, symptom profiles, medication status, and baseline neurophysiological characteristics, rather than inconsistency in the effects of exercise alone (Zhou et al., 2026). As the evidence base expands, future meta-analyses should, where possible, conduct more granular subgroup or meta-regression analyses according to specific clinical conditions, symptom profiles, medication status, and other relevant participant characteristics.

#### 4.2.3 Analysis of acute and chronic exercise

With respect to the exercise intervention paradigm, the pooled effect estimates were positive for both the acute- and chronic-exercise subgroups. The pooled point estimate was numerically larger for acute exercise than for chronic exercise; however, the estimate for acute exercise was imprecise, with a wide confidence interval that included zero, and was not statistically significant. By contrast, the pooled estimate for chronic exercise was statistically significant, although its confidence interval was also relatively wide and its lower bound was close to zero. Nevertheless, the formal test for subgroup differences was not statistically significant, providing no evidence that the effects of acute and chronic exercise on EEG alpha-band power differed. Accordingly, the numerically larger point estimate for acute exercise should not be interpreted as evidence that acute exercise produces a greater effect than chronic exercise. Acute and chronic exercise may affect EEG alpha-band activity through partly distinct neurophysiological mechanisms (Petruzzello et al., 1991). Acute exercise may elicit transient neurophysiological responses during or shortly after a single exercise session, including changes in arousal, cerebral blood flow, autonomic regulation, and neurotransmitter activity (Moritani et al., 1997). By contrast, repeated exercise training may produce more sustained adaptations involving neuroplasticity and longer-term functional reorganization (El-Sayes et al., 2019). Despite these plausible mechanistic differences, the available evidence directly comparing acute and chronic exercise remains limited. Moreover, considerable heterogeneity was observed within the acute-exercise subgroup, and the timing of postexercise EEG assessment varied across studies. Together with the small number of studies, these methodological differences may have reduced the power of the subgroup comparison to detect a true difference between acute and chronic exercise.

#### 4.2.4 Differences in EEG equipment and data processing

EEG is a noninvasive neurophysiological recording technique with high temporal resolution and has been widely used in cognitive neuroscience, clinical neurophysiology, and brain–computer interface research (Islam et al., 2016). However, EEG signals are low in amplitude and are highly susceptible to contamination by artifacts arising from both physiological and nonphysiological sources. Consequently, preprocessing is a critical component of EEG analysis because it is used to attenuate artifacts and improve the signal-to-noise ratio. At the same time, variation in preprocessing pipelines—including artifact correction, filtering, segmentation, referencing, and spectral estimation—can introduce substantial methodological heterogeneity and affect the comparability of EEG alpha-power estimates across studies. Differences in EEG hardware and acquisition settings, including channel density, electrode montage, and sampling rate, may contribute to variation in study findings. Preprocessing procedures are another important source of methodological variability. Although independent component analysis is widely used to attenuate ocular and cardiac artifacts, its performance depends on data quality and component-selection decisions, potentially introducing analyst-related variability (Leach et al., 2020). Reference schemes, filter settings, frequency-band definitions, and spectral estimation methods can also alter the magnitude and distribution of estimated EEG alpha-band power (Bigdely-Shamlo et al., 2015). Collectively, variation in EEG acquisition and processing procedures may have contributed to the considerable between-study heterogeneity observed in this meta-analysis, underscoring the need for more standardized and transparent methodological reporting.

Overall, the prespecified subgroup analyses did not explain the considerable between-study heterogeneity, suggesting that the observed variation may reflect the combined influence of multiple unmeasured or insufficiently characterized factors. Potential sources include exercise modality, intensity, frequency, and dose, as well as participant characteristics and baseline neurophysiological status. Previous evidence suggests that exercise parameters may differentially influence cortical activity (Ludyga et al., 2016). Variation in EEG hardware, electrode montage, preprocessing, spectral estimation, and statistical analysis may also influence alpha-power estimates and reduce cross-study comparability. Future studies should therefore adopt more standardized protocols and provide sufficiently detailed reporting of exercise prescriptions, participant characteristics, and EEG acquisition and processing procedures.

### 4.3 EEG recording conditions, intervention duration, and result robustness

Despite the considerable between-study heterogeneity, the direction and statistical significance of the pooled effect estimate appeared reasonably robust across the sensitivity analyses. First, the leave-one-out sensitivity analysis showed that the pooled effect estimate remained positive and statistically significant after each study was excluded in turn, indicating that the overall finding was not driven by any single study. Nevertheless, this robustness primarily concerned the direction and statistical significance of the pooled estimate and did not eliminate the uncertainty arising from the substantial heterogeneity across studies.

Second, sensitivity analyses based on EEG recording conditions provided additional insight into potential methodological sources of heterogeneity. As the analysis was progressively restricted from all included studies to resting-state studies and then to resting-state studies that explicitly reported whether recordings were obtained with eyes open or closed, between-study heterogeneity decreased stepwise (*I^2^* = 91.56, 86.71, and 69.46%, respectively), while the pooled effect estimate remained statistically significant in each analysis. This pattern suggests that variation in EEG recording conditions may have contributed to the observed heterogeneity. Resting-state and task-based recordings capture different neurophysiological states, and eyes-open and eyes-closed conditions may also influence EEG alpha-band power and its scalp distribution (Greicius et al., 2003). Pooling effect estimates across these recording conditions may therefore increase methodological variability and reduce cross-study comparability (Gonçalves et al., 2006). However, because the studies retained at each stage may also have differed in participant characteristics, intervention protocols, and other methodological features, these sensitivity analyses cannot establish EEG recording condition as a formal moderator. More definitive conclusions will require adequately powered subgroup analyses or multivariable meta-regression once a sufficient number of studies becomes available.

Beyond recording conditions, an exploratory univariable meta-regression restricted to the eight studies of chronic exercise examined whether intervention duration was associated with effect magnitude. The analysis yielded a positive slope, indicating that the estimated effect size tended to increase with each additional week of intervention; however, this linear association was not statistically significant. Although intervention duration accounted for approximately 26.81% of the between-study heterogeneity, considerable residual heterogeneity remained in the model. Accordingly, the available evidence does not establish a clear linear dose–response association between the duration of chronic exercise interventions and their effects on EEG alpha-band power. Several factors may explain this inconclusive result. First, the small number of studies limited statistical power and increased the sensitivity of the estimate to individual studies (Bizuayehu et al., 2024). Second, intervention duration in weeks does not fully capture exercise dose, because studies of the same overall duration may differ in session frequency, session duration, exercise intensity, and adherence. Third, longer interventions may not necessarily produce proportionally larger neurophysiological effects, because exercise-related adaptations may follow nonlinear trajectories or reach a plateau over time (Xue et al., 2019).

The influence diagnostics further reinforced the need for caution. Several individual studies exerted substantial influence on both the estimated meta-regression slope and the residual heterogeneity. Although the direction of the slope remained positive when influential studies were excluded one at a time, its magnitude varied across analyses, indicating that the estimate was not highly stable. The observed association between intervention duration and EEG alpha-band power should therefore be regarded as exploratory rather than confirmatory and does not provide sufficient evidence to conclude that extending the duration of exercise training causes greater increases in EEG alpha-band power. Taken together, the overall pooled effect appeared reasonably robust to the exclusion of individual studies and to restriction by EEG recording condition, but the sources of heterogeneity and the potential duration–effect relationship remain uncertain.

### 4.4 Significance, limitations and future perspectives of the research

#### 4.4.1 Significance of the research

This review provides preliminary quantitative evidence that exercise interventions may alter EEG alpha-band power. The positive pooled effect remained statistically significant across multiple sensitivity analyses, suggesting that the observed increase in EEG alpha-band power was not driven solely by any single study or by the inclusion of a particular recording condition. These findings provide a quantitative basis for further investigation of how exercise-related modulation of EEG alpha-band activity may relate to neurophysiological regulation, cognitive functioning, and clinical symptom management. For clinicians and rehabilitation professionals, the findings support continued consideration of exercise as a potentially relevant adjunctive strategy, while also underscoring that the present evidence is insufficient to justify specific clinical recommendations based on EEG alpha-band outcomes alone (Chen and Lu, 2025; Wan et al., 2024). Moreover, the considerable between-study heterogeneity precludes firm conclusions regarding the optimal exercise modality, intensity, frequency, or duration.

#### 4.4.2 Limitations of the research

This study has several limitations. Most importantly, the considerable between-study heterogeneity limits the generalizability of the pooled effect estimate and indicates that the magnitude of the effect may vary substantially across study settings. Second, the relatively small number of included studies limited the statistical power of the subgroup analyses and meta-regression and reduced the reliability of assessments of small-study effects and publication bias. Although Egger’s regression test was not statistically significant, its power was likely limited in the presence of few studies and considerable heterogeneity. Accordingly, small-study effects and the selective nonpublication of null or unfavorable findings cannot be ruled out. Third, the included studies varied substantially in exercise modality, intensity, frequency, duration, and participant characteristics. Because these variables were incompletely or inconsistently reported, their potential contributions to between-study heterogeneity could not be examined in greater detail. Some studies also reported alpha-power estimates for multiple scalp regions, electrodes, or analytic outcomes. Variation in the selection of regions and outcome measures may therefore have affected the pooled estimate and introduced additional outcome-selection variability. Finally, differences in EEG acquisition, preprocessing, referencing, spectral estimation, and alpha-power calculation may have introduced additional methodological heterogeneity and reduced cross-study comparability.

#### 4.4.3 Future perspectives of the research

Based on the findings and limitations of the present meta-analysis, future research should prioritize the following directions:

1. Adequately powered, high-quality randomized controlled trials are needed. Future studies should recruit larger samples, follow established reporting guidelines such as CONSORT, and provide detailed descriptions of exercise modality, intensity, frequency, session duration, total intervention duration, adherence, and participant characteristics. More complete and consistent reporting would facilitate replication, improve the quality of future evidence syntheses, and support the eventual development of more individualized exercise recommendations.
2. Future studies should focus on more clearly defined populations and evaluate the persistence of intervention effects. Recruiting clinically and demographically more homogeneous samples would help clarify whether exercise-related changes in EEG alpha-band power differ across specific populations and clinical conditions (Ben Ezzdine et al., 2025). Longer postintervention follow-up is also needed to determine whether observed neurophysiological changes persist after structured exercise training has ended.
3. The dose–response relationship should be examined using prospectively designed studies. Future trials should systematically compare exercise intensity, frequency, session duration, total training volume, and overall intervention duration. These studies should also consider potential nonlinear, threshold, or plateau effects rather than assuming a simple linear relationship. Identifying the minimum effective dose and the range of doses associated with the most favorable EEG alpha-band responses would provide a stronger empirical basis for developing precise exercise prescriptions (Gomes-Osman et al., 2018; Jayedi et al., 2024).
4. Complementary EEG analysis methods should be incorporated where appropriate. In addition to conventional spectral power analysis, future studies may use time–frequency analysis, functional connectivity, source-space analysis, and network topology to characterize exercise-related changes in neural dynamics more comprehensively (Peng et al., 2026; Wu et al., 2026). These methods should be selected according to clearly defined research questions, prespecified where possible, and accompanied by appropriate control of analytic flexibility and multiple testing.
5. EEG acquisition and processing protocols require greater standardization. Future studies should clearly report the recording state, including whether EEG was obtained at rest or during a task, whether participants’ eyes were open or closed, and the timing of assessment relative to exercise. Electrode montage, channel density, sampling rate, reference scheme, filtering parameters, artifact-handling procedures, epoch selection, alpha-band definition, spectral estimation method, and the calculation of absolute or relative power should also be documented in sufficient detail. Greater methodological standardization and transparent reporting would reduce avoidable variability and improve reproducibility and comparability across studies.

## 5 Conclusion

This meta-analysis suggests that exercise interventions may increase EEG alpha-band power. The direction and statistical significance of the pooled effect estimate remained stable across sensitivity analyses, indicating that the overall finding was not driven by any single study or solely by the inclusion of a particular recording condition. Nevertheless, considerable between-study heterogeneity and a prediction interval that included zero indicate that the magnitude, and potentially the presence, of the true effect may vary across study settings. No evidence was found that the EEG alpha-power metric, population type, or exercise intervention paradigm moderated the pooled effect. The stepwise reduction in heterogeneity after restricting the analysis to studies with more comparable EEG recording conditions suggests that resting versus task-based recordings and eyes-open versus eyes-closed protocols may have contributed to the observed variability, although this interpretation requires confirmation through formal moderator analyses. Overall, EEG alpha-band power appears to be a context-dependent electrophysiological outcome that may be sensitive to exercise-related changes under some study conditions, but the observed changes should not be interpreted as evidence of cognitive or clinical improvement. Confidence in the pooled estimate remains limited by risk-of-bias concerns, inconsistency, imprecision, and methodological diversity. Future adequately powered trials should standardize exercise prescriptions and EEG acquisition and processing procedures and determine whether exercise-related changes in EEG alpha-band power are associated with meaningful and sustained functional outcomes.

## Data Availability

The raw data supporting the conclusions of this article will be made available by the authors, without undue reservation.
