# Supplementary material for: Effects of exercise interventions on EEG alpha power across different populations: a systematic review and meta-analysis
Source: Front Psychol. 2026 Aug 6;17:1752573. doi: 10.3389/fpsyg.2026.1752573 (PMC13489911; doi:10.3389/fpsyg.2026.1752573)
Supplement: Supplementary file 1 [file Table_1.docx]

**Search formula**

| Database | **Search Results Summary** | **1369** |
| --- | --- | --- |
| PubMed： | **((("Exercise"[Mesh] OR "Exercise Therapy"[Mesh] OR "Athletic Performance"[Mesh] OR "Yoga"[Mesh] OR "Tai Ji"[Mesh] OR "High-Intensity Interval Training"[Mesh] OR exercis*[tiab] OR aerobic[tiab] OR "resistance training"[tiab] OR "strength training"[tiab] OR "physical activity"[tiab] OR "HIIT"[tiab] OR yoga[tiab] OR "tai chi"[tiab])) AND (("Electroencephalography"[Mesh] OR EEG[tiab] OR "electroencephalogram"[tiab] OR "electroencephalographic"[tiab] OR "brain waves"[tiab] OR "neural activity"[tiab])) AND ("power spectrum"[tiab] OR "spectral power"[tiab] OR "alpha power"[tiab] OR "beta power"[tiab] OR "theta power"[tiab] OR "delta power"[tiab] OR "gamma power"[tiab] OR "band power"[tiab])** | **282** |
| Embase： | **'exercise'/exp OR 'exercise therapy'/exp OR 'yoga'/exp OR 'high intensity interval training'/exp OR 'tai chi'/exp OR (exercis* OR aerobic OR 'resistance training' OR 'strength training' OR 'physical activity' OR hiit OR yoga OR 'tai chi' OR 'tai ji'):ti,ab,kw AND 'electroencephalography'/exp OR (eeg OR electroencephalogram OR electroencephalography OR 'brain waves' OR 'neural activity'):ti,ab,kw**  **AND ('power spectrum' OR 'spectral power' OR 'alpha power' OR 'beta power' OR 'theta power' OR 'delta power' OR 'gamma power' OR 'band power'):ti,ab,kw** | **312** |
| Web of science： | **(exercis* OR aerobic OR "resistance training" OR "strength training" OR "physical activity" OR HIIT OR yoga OR "tai chi" OR "tai ji" OR "high intensity interval training")**  **AND (EEG OR electroencephalogram OR electroencephalography OR "brain waves" OR "neural activity") AND ("power spectrum" OR "spectral power" OR "alpha power" OR "beta power" OR "theta power" OR "delta power" OR "gamma power" OR "band power")** | **249** |
| Cochrane： | **(exercise OR "physical activity" OR "aerobic exercise" OR "resistance training" OR "strength training" OR "high-intensity interval training" OR yoga OR "tai chi" OR sport* OR running OR walking OR cycling) Title, Abstract, Keywords AND (EEG OR electroencephalogram OR electroencephalography OR "brain waves" OR "neural activity") Title, Abstract, Keywords AND ("power spectrum" OR "spectral power" OR "alpha power" OR "beta power" OR "theta power" OR "delta power" OR "gamma power" OR "band power") Title, Abstract, Keywords** | **128** |
| Scopus： | **( exercis* OR aerobic OR "resistance training" OR "strength training" OR "physical activity" OR HIIT OR yoga OR "tai chi" OR "tai ji" OR "high intensity interval training" )AND TITLE-ABS-KEY ( eeg OR electroencephalogram OR electroencephalography OR "brain waves" OR "neural activity" ) ) ) AND ( TITLE-ABS-KEY ( "power spectrum" OR "spectral power" OR "alpha power" OR "beta power" OR "theta power" OR "delta power" OR "gamma power" OR "band power" ) )** | **398** |

| First author/Year | Number /Age（M ± SD） | | band | Experimental | | | | | | | Control | | | | | | | Intervention programme | cycle | Time | Frequency  /week |
| --- | --- | --- | --- | --- | --- | --- | --- | --- | --- | --- | --- | --- | --- | --- | --- | --- | --- | --- | --- | --- | --- |
|  |  |  |  | **Pre** | | **Later** | | **meta** | | | **Pre** | | **Later** | | **meta** | | |  |  |  |  |
|  | Experimental | Control |  | Mean | SD | Mean | SD | N1 | SMD1 | SD1 | Mean | SD | Mean | SD | N1 | SMD1 | SD1 |  |  |  |  |
| Du Rietz, E.2019 | 13人 21.5±2.52 | 13人 21.5±2.52 | α | 0.830 | 1.030 | 1.060 | 1.130 | 13 | 0.223 | 0.290 | 0.940 | 1.150 | 1.110 | 1.160 | 13 | 0.148 | 0.290 | cycling | acute | 30min | single |
| Hung, T. M.2018 | 24人 9.54 ± 1.59 | 28人 9.96 ± 1.11 | α | 2.030 | 0.300 | 2.060 | 0.410 | 24 | 0.100 | 0.209 | 1.790 | 0.350 | 1.860 | 0.350 | 28 | 0.200 | 0.197 | running | acute | 30min | single |
| Kim, S. R.2015 | 10人 69.5 ± 3.2 | 10人 69.7 ± 3.5 | α | 0.064 | 0.014 | 0.071 | 0.014 | 10 | 0.500 | 0.354 | 0.053 | 0.012 | 0.047 | 0.011 | 10 | -0.500 | 0.354 | Equestrian | 8weeks | 15min | 3times |
| Nagendra, H.2015 | 15人 22.42 ± 2.30 | 15人 23.67 ± 2.09 | α | 3.950 | 0.700 | 5.670 | 1.680 | 15 | 2.457 | 0.676 | 3.920 | 0.970 | 3.870 | 0.900 | 15 | -0.052 | 0.262 | Yoga | 5months | 90min | 6times |
| Rathi, G.2025 | 15人 19.31 ± 1.33 | 19人 19.8 ± 0.97 | α | 0.054 | 0.016 | 0.100 | 0.035 | 15 | 2.968 | 0.600 | 0.027 | 0.009 | 0.034 | 0.012 | 19 | 0.778 | 0.262 | Yoga postures | 24weeks | 30min | 2times |
| Sung-Hyoun Cho2017 | 15人 67.6 ± 2.32 | 16人 67.88 ± 2.39 | α | 0.063 | 0.040 | 0.070 | 0.040 | 15 | 0.175 | 0.267 | 0.052 | 0.035 | 0.046 | 0.031 | 16 | -0.171 | 0.259 | Equestrian | 12weeks | 25min | 2times |
| Wang, M.2024 | 35人 20.08 ± 0.28 | 35人 20.71 ± 0.51 | α | 5.170 | 4.060 | 5.800 | 4.670 | 35 | 0.155 | 0.175 | 5.380 | 4.020 | 2.640 | 1.440 | 35 | -0.681 | 0.181 | Tai Chi | 12weeks | 45min | 3times |
| Wang, M.2025 | 22人 20.09 ± 0.29 | 23人 20.70 ± 0.56 | α | 8.790 | 15.130 | 11.140 | 16.730 | 22 | 0.155 | 0.226 | 7.500 | 8.460 | 3.500 | 2.830 | 23 | -0.473 | 0.218 | Tai Chi | 12weeks | 45min | 3times |
| Yu-Joung Son2017 | 11人 40.81 ± 12.1 | 10人 36.9 ± 10.71 | α | 0.319 | 0.052 | 0.235 | 0.042 | 11 | -1.615 | 0.530 | 0.331 | 0.034 | 0.302 | 0.044 | 10 | -0.853 | 0.358 | Pilates | 6weeks | 50min | 3times |
| Hao Sun.2026 | 28人18.00±0.77 | 28人18.04±0.84 | α | 37.55 | 18.2 | 50.77 | 16.64 | 28 | 0.726 | 0.207 | 37.90 | 13.06 | 36.73 | 14.98 | 28 | -0.090 | 0.205 | VR boxing | acute | 30min | single |
| Tomomi Nomura.2026 | 20人24.2±4.4 | 20人25.9±5.5 | α | 2.563 | 2.397 | 3.583 | 3.244 | 20 | 0.426 | 0.281 | 1.526 | 3.014 | 1.672 | 2.692 | 20 | 0.048 | 0.213 | Cycling | acute | 20min | single |
| Huipeng Lei.2026 | 16人19.00±1.83 | 16人20.19±2.13 | α | 0.317 | 0.020 | 0.397 | 0.013 | 16 | 4.000 | 0.763 | 0.343 | 0.013 | 0.380 | 0.023 | 16 | 2.846 | 0.646 | HIIT | 8weeks | 30min | 3times |
| Tongtong Che.2026 | 20人21.58±2.21 | 20人20.81±2.52 | α | 0.02957 | 0.00221 | 0.03354 | 0.00256 | 20 | 1.796 | 0.380 | 0.02877 | 0.00171 | 0.03054 | 0.00185 | 20 | 1.035 | 0.287 | Cycling | acute | 20min | single |

**Document inclusion consent rate**

| Screening stage | Both are included | Both are excluded | Disagreement | consent rate | Kappa |
| --- | --- | --- | --- | --- | --- |
| Title/Abstract (n=727) | 130 | 575 | 22 | 96.97% | 0.903 |
| Full text (n=145) | 18 | 121 | 6 | 95.86% | 0.833 |

**Detailed results of Cochrane's risk of bias assessment**

| Number | First Author/ Year | Randomization process | Deviation from the expected intervention | Lack of ending data | Outcome measurement | Selective reporting of results | Overall bias risk |
| --- | --- | --- | --- | --- | --- | --- | --- |
| 1 | Du Rietz, E.2019 | Some concerns | Low | Some concerns | Low | Some concerns | Some concerns |
| 2 | Hung, T. M.2018 | Some concerns | Low | Low | Low | Some concerns | Some concerns |
| 3 | Kim, S. R.2015 | Some concerns | Low | Low | Some concerns | Some concerns | Some concerns |
| 4 | Nagendra, H.2015 | Low | Low | Low | Some concerns | Some concerns | Some concerns |
| 5 | Rathi, G.2025 | Low | Low | Low | Some concerns | High | High |
| 6 | Sung-Hyoun Cho2017 | Some concerns | Low | Low | Some concerns | Some concerns | Some concerns |
| 7 | Wang, M.2024 | Some concerns | Low | Low | Low | Some concerns | Some concerns |
| 8 | Wang, M.2025 | Some concerns | Low | High | Low | Low | High |
| 9 | Yu-Joung Son2017 | Some concerns | Low | High | Some concerns | Some concerns | High |
| 10 | Hao Sun.2026 | Some concerns | Low | Low | Some concerns | Some concerns | Some concerns |
| 11 | Tomomi Nomura.2026 | Low | Low | Low | Low | Some concerns | Some concerns |
| 12 | Huipeng Lei.2026 | Low | Low | Low | Some concerns | Some concerns | Some concerns |
| 13 | Tongtong Che.2026 | Some concerns | Low | Low | Low | Some concerns | Some concerns |

Extraction of fundamental characteristics from EEG data

| First author/Year | Filters | | Artifact handling | Alpha-band definition | Spectral estimation | Electrodes/ROI |
| --- | --- | --- | --- | --- | --- | --- |
| Du Rietz, E.2019 | | Downsampled from 500 to 256 Hz; offline 0.1–30 Hz digital band-pass Butterworth filter (24 dB/oct). | Visual inspection and manual removal; ICA correction of ocular components; automatic rejection when any channel exceeded ±100 μV; removed channels replaced by topographic spline interpolation. | 7.5–12.5 Hz | FFT on artifact-free 2-s epochs. | Frontal ROI: Fz, F1–F8; central ROI: Cz, C1–C6; parietal ROI: Pz, P3–P8. |
| Hung, T. M.2018 | | Acquisition bandwidth DC–100 Hz with 60 Hz notch; offline 1–30 Hz band-pass filter (12 dB/oct). | EOG correction by an unspecified algorithm; visual artifact inspection; cleaned data segmented into 2-s epochs. | 8–12.5 Hz | FFT; mean power estimates from 2-s epochs, followed by natural-log transformation. | Midline electrodes Fz, Cz, Pz. |
| Kim, S. R.2015 | | NA | Movement, chewing, and talking were minimized during recording; the first and last 60 s were excluded. No post-recording artifact-removal algorithm or rejection threshold was reported. | 8–13 Hz; | FFT followed by power-spectrum and band-to-band power analysis. | Fp1, Fp2, F3, F4, T3, T4, P3, P4. |
| Nagendra, H.2015 | | No conventional digital band-pass/notch filter was reported. In the wavelet decomposition, D1–D3 were treated as noise components and excluded; the stated useful EEG range was 0.5–70 Hz. | Participants minimized blinking and movement; recording was prolonged when artifacts occurred; EEG was manually edited and artifact-free data were selected. | Nominal alpha definition: 8–13 Hz. | Seven-level discrete wavelet transform using db4; band power/PSD derived from wavelet coefficients. | frontal, central, parietal, occipital, and temporal lobes |
| Rathi, G.2025 | | Linear detrending (SciPy) and resampling were reported. No explicit conventional band-pass or notch-filter settings were reported. | Eye blinks, eye movements, and muscle activity were identified and removed by visual inspection, automated algorithms, or both; the exact algorithm and rejection threshold were not specified. | 8–13 Hz | Welch PSD estimation: overlapping segments, windowing, periodogram calculation, and averaging. | P3/P4, F3/F4, T5/T6, O1/O2 |
| Sung-Hyoun Cho2017 | | NA | Movement, chewing, and speaking were minimized; the initial and final 60 s were excluded. No post-recording artifact-removal algorithm or rejection threshold was reported. | 8–13 Hz | FFT followed by power-spectrum and band-to-band power analysis. | Fp1, Fp2, F3, F4, T3, T4, P3, P4. |
| Wang, M.2024 | | 50 Hz notch; IIR high-pass 0.5 Hz and low-pass 40 Hz; average re-reference. | Semiautomatic artifact inspection and removal; only 2-s artifact-free epochs were retained. | 8–12 Hz | FFT and PSD analysis on 2-s artifact-free epochs; values averaged per participant. | F3 and F4 |
| Wang, M.2025 | | 50 Hz notch; IIR high-pass 0.5 Hz and low-pass 40 Hz; average re-reference. | Semiautomatic inspection using gradient, maximum–minimum, and amplitude criteria; bad intervals skipped; 2-s artifact-free epochs retained. | 8–12 Hz | FFT for each 2-s segment; PSD values averaged per participant. | F3, F4, C3, C4, P3, P4, T7, T8. |
| Yu-Joung Son2017 | | NA | NA | 8–12.99 Hz | Relative power-spectrum analysis in TeleScan; the spectral algorithm, epoch length, and window were not reported. | Fp1, Fp2, F3, F4, C3, C4, O1, O2 |
| Hao S.2026 | | Downsampled to 250 Hz; 0.1–30 Hz band-pass filter; 50 Hz notch filter. | Automatic rejection of 2-s epochs exceeding ±100 μV; visual removal of residual muscle, blink, and electrode artifacts; ICA removal of ocular and persistent muscle components; persistently faulty channels excluded. | 8–13 Hz | Fourier transform of successive non-overlapping 2-s epochs; window and exact PSD normalization were not reported. | Fp1, Fp2, F3, F4. Left frontal aggregate: Fp1+F3; right frontal aggregate: Fp2+F4. |
| Tomomi N.2026 | | Acquisition and digital IIR band-pass filter: 0.5–70 Hz. | Epoch-wise baseline correction; rigorous visual inspection; 60 s of artifact-free data manually selected for each participant. | 7.84–13.23 Hz | FFT using a 2-s Hanning window, zero-padding, and 0% overlap; 30 segments per participant. | F3, F4, C3, C4, P3, P4, O1, O2. |
| Huipeng L.2026 | | 1–50 Hz Butterworth band-pass filter; 50 Hz notch filter; re-referenced to common average. | Visual removal of gross movement/electrode-drift segments; ICA removal of ocular, cardiac, and muscle components; persistently noisy channels interpolated with spherical splines; residual artifacts rejected using automated amplitude screening plus visual inspection. | 8–13 Hz | Welch PSD on 2-s epochs. | Frontal ROI: F3/Fz/F4; central ROI: C3/Cz/C4; posterior ROI: P3/Pz/P4. |
| Tongtong C.2026 | | Hardware 0.01–100 Hz band-pass and 50 Hz notch; offline 1–40 Hz band-pass. | ICA removal of eye-movement and blink components. No amplitude threshold, channel interpolation, or manual rejection rule was reported. | 8–13 Hz | FFT used to calculate PSD; data were segmented by experimental phase, but epoch length, overlap, and window were not reported. | prefrontal Fp1/Fp2/Fz, central C3/C4/Cz, parietal P3/P4. |

R：# 1. 安装并加载包 ----------------------------------------------------------

packages <- c("readxl", "dplyr", "metafor")

new_packages <- packages[!vapply(packages, requireNamespace, logical(1), quietly = TRUE)]

if (length(new_packages) > 0) install.packages(new_packages)

library(readxl)

library(dplyr)

library(metafor)

\# 2. 读取 Excel ------------------------------------------------------------

\# 运行后选择“新建 XLSX 工作表.xlsx”

file_path <- file.choose()

raw_dat <- read_excel(file_path, sheet = "Sheet1", .name_repair = "unique")

\# 当前第10列没有表头，但内容为 RCT；按列位置统一重命名

if (ncol(raw_dat) < 9) {

stop("表格至少需要9列：number、study、year、n1、均值1、sd1、n2、均值2、sd2。")

}

if (ncol(raw_dat) >= 10) {

dat <- raw_dat[, 1:10]

names(dat) <- c(

"number", "study", "year",

"n1", "mean1", "sd1",

"n2", "mean2", "sd2",

"design"

)

} else {

dat <- raw_dat[, 1:9]

names(dat) <- c(

"number", "study", "year",

"n1", "mean1", "sd1",

"n2", "mean2", "sd2"

)

dat$design <- NA_character_

}

\# 去除尾部空白行并转换变量类型

dat <- dat %>%

filter(!is.na(study)) %>%

mutate(

number = as.numeric(number),

study = as.character(study),

year = as.numeric(year),

n1 = as.numeric(n1),

mean1 = as.numeric(mean1),

sd1 = as.numeric(sd1),

n2 = as.numeric(n2),

mean2 = as.numeric(mean2),

sd2 = as.numeric(sd2),

design = as.character(design),

study_label = paste0(study, " (", year, ")")

)

\# 3. 数据检查 --------------------------------------------------------------

required <- c("n1", "mean1", "sd1", "n2", "mean2", "sd2")

if (any(!complete.cases(dat[, required]))) {

stop("研究数据中存在缺失值，请检查 n、均值和 SD。")

}

if (any(dat$n1 < 2 | dat$n2 < 2)) {

stop("发现样本量小于2的研究。")

}

if (any(dat$sd1 <= 0 | dat$sd2 <= 0)) {

stop("发现 SD 小于或等于0的研究。")

}

\# 重要：确认 sd1/sd2 是标准差 SD，而不是标准误 SE。

\# 若文献报告 SE，应换算：SD = SE * sqrt(n)

\# 4. 统一效应方向 ----------------------------------------------------------

\# 正值表示实验组的 alpha 波变化大于对照组，即实验组效果更好。

\# 当前所有研究若均采用“变化值越大越好”的编码，则不需要反向。

\# 只有原始文献对变化值采用相反编码时，才把对应研究编号填入下方。

flip_numbers <- c()

dat <- dat %>%

mutate(direction = ifelse(number %in% flip_numbers, -1, 1))

\# 结果方向解释：

\#	Hedges' g > 0：实验组 alpha 波变化更大，支持实验干预；

\#	Hedges' g = 0：两组无差异；

\#	Hedges' g < 0：对照组 alpha 波变化更大。

\#

\# 注意：只有在各研究的“变化值”计算方向一致时才能直接合并。

\# 例如，所有研究都应尽量统一为：后测值 - 前测值。

\# 若某研究使用前测值 - 后测值，需要先将该研究的均值乘以 -1，

\# 或将该研究编号加入 flip_numbers。

\# 5. 计算标准化均数差 Hedges' g --------------------------------------------

es <- escalc(

measure = "SMD",

m1i = mean1,

sd1i = sd1,

n1i = n1,

m2i = mean2,

sd2i = sd2,

n2i = n2,

data = dat,

vtype = "LS",

slab = study_label

)

\# 反向编码只改变效应值符号，方差不变

es$yi <- es$yi * es$direction

es$sei <- sqrt(es$vi)

cat("\n================ 各研究 Hedges' g ================\n")

print(es[, c("number", "study_label", "yi", "sei")], digits = 4)

\# 标记绝对值特别大的效应量，优先核查 SD/SE、方向和录入

large_effects <- es[abs(es$yi) > 2, c("number", "study_label", "yi", "mean1", "sd1", "mean2", "sd2")]

if (nrow(large_effects) > 0) {

cat("\n========== |Hedges' g| > 2，需要重点核查 ==========\n")

print(large_effects, digits = 4)

}

\# 6. 主分析：随机效应模型 REML + Knapp-Hartung -----------------------------

res <- rma(

yi = yi,

vi = vi,

data = es,

method = "REML",

test = "knha"

)

cat("\n================ 主分析结果 ================\n")

print(res, digits = 4)

cat("\n================ 异质性区间 ================\n")

print(confint(res), digits = 4)

cat("\n================ 预测区间 ================\n")

pred <- predict(res)

print(pred, digits = 4)

\# 7. 创建结果文件夹 --------------------------------------------------------

out_dir <- file.path(dirname(file_path), "meta_results")

dir.create(out_dir, showWarnings = FALSE, recursive = TRUE)

\# 8. 森林图 ---------------------------------------------------------------

pdf(file.path(out_dir, "01_forest_plot.pdf"), width = 9.5, height = 8.5)

forest(

res,

slab = es$study_label,

xlab = "Hedges' g（正值表示实验组 alpha 波改善优于对照组）",

mlab = "随机效应模型：REML + Knapp-Hartung",

addpred = TRUE,

header = c("研究", "Hedges' g [95% CI]")

)

abline(v = 0, lty = 2)

dev.off()

\# 9. 敏感性分析：逐一剔除 -------------------------------------------------

loo <- leave1out(res)

cat("\n================ Leave-one-out ================\n")

print(loo, digits = 4)

write.csv(

as.data.frame(loo),

file.path(out_dir, "02_leave_one_out.csv"),

row.names = FALSE,

fileEncoding = "UTF-8"

)

\# 10. 影响诊断 -------------------------------------------------------------

inf <- influence(res)

cat("\n================ 影响诊断 ================\n")

print(inf, digits = 4)

pdf(file.path(out_dir, "03_influence_diagnostics.pdf"), width = 10, height = 8)

plot(inf, slab.style = 3, las = 3)

dev.off()

\# 只将诊断结果作为核查依据，不自动删除研究

capture.output(

print(inf, digits = 4),

file = file.path(out_dir, "03_influence_diagnostics.txt")

)

\# 11. Baujat 图：识别对 Q 和合并效应贡献较大的研究 -------------------------

pdf(file.path(out_dir, "04_baujat_plot.pdf"), width = 8, height = 7)

baujat(res)

dev.off()

\# 12. 不同 tau^2 估计方法的稳健性分析 --------------------------------------

tau_methods <- c("REML", "PM", "DL", "SJ")

estimator_sensitivity <- do.call(

rbind,

lapply(tau_methods, function(meth) {

fit <- rma(

yi = yi,

vi = vi,

data = es,

method = meth,

test = "knha"

)

pr <- predict(fit)

data.frame(

method = meth,

k = fit$k,

estimate = as.numeric(coef(fit)),

ci_lb = as.numeric(fit$ci.lb),

ci_ub = as.numeric(fit$ci.ub),

p_value = as.numeric(fit$pval),

tau2 = as.numeric(fit$tau2),

I2_percent = as.numeric(fit$I2),

prediction_lb = as.numeric(pr$pi.lb),

prediction_ub = as.numeric(pr$pi.ub)

)

})

)

cat("\n========== tau^2 估计方法敏感性分析 ==========\n")

print(estimator_sensitivity, digits = 4)

write.csv(

estimator_sensitivity,

file.path(out_dir, "05_tau2_method_sensitivity.csv"),

row.names = FALSE,

fileEncoding = "UTF-8"

)

\# 13. 漏斗图和 Egger 检验：仅作小样本效应辅助分析 --------------------------

pdf(file.path(out_dir, "06_funnel_plot.pdf"), width = 8, height = 7)

funnel(res, yaxis = "sei")

dev.off()

egger <- regtest(res, model = "rma", predictor = "sei")

cat("\n================ Egger 检验 ================\n")

print(egger, digits = 4)

capture.output(

print(egger, digits = 4),

file = file.path(out_dir, "06_egger_test.txt")

)

\# 14. 导出主要结果 ---------------------------------------------------------

main_result <- data.frame(

k = res$k,

pooled_Hedges_g_experimental_vs_control = as.numeric(coef(res)),

ci_lb = as.numeric(res$ci.lb),

ci_ub = as.numeric(res$ci.ub),

p_value = as.numeric(res$pval),

Q = as.numeric(res$QE),

Q_df = res$k - res$p,

Q_p_value = as.numeric(res$QEp),

tau2 = as.numeric(res$tau2),

tau = sqrt(as.numeric(res$tau2)),

I2_percent = as.numeric(res$I2),

prediction_lb = as.numeric(pred$pi.lb),

prediction_ub = as.numeric(pred$pi.ub)

)

write.csv(

as.data.frame(es),

file.path(out_dir, "00_effect_sizes.csv"),

row.names = FALSE,

fileEncoding = "UTF-8"

)

write.csv(

main_result,

file.path(out_dir, "00_main_result.csv"),

row.names = FALSE,

fileEncoding = "UTF-8"

)

capture.output(sessionInfo(), file = file.path(out_dir, "99_sessionInfo.txt"))

cat("\n============================================================\n")

cat("分析完成。结果保存在：\n", normalizePath(out_dir), "\n")

cat("报告时至少包括：Hedges' g、95% CI、Q、I²、tau²和预测区间。\n")

cat("不要仅为了降低 I² 而删除研究；应根据数据错误或明确的方法学理由处理。\n")

cat("============================================================\n")
